# Examining Macro-Level Barriers and Facilitators to Scaling Up Integrated Care from a Complexity Perspective: A Multi-Case Study of Cambodia, Slovenia, and Belgium

**DOI:** 10.5334/ijic.7650

**Published:** 2024-11-12

**Authors:** Monika Martens, Savina Chham, Zavrnik Črt, Katrien Danhieux, Edwin Wouters, Srean Chhim, Antonija Poplas Susič, Zalika Klemenc Ketiš, Por Ir, Roy Remmen, Kerstin Klipstein-Grobusch, Wim Van Damme, Grace Marie Ku, Josefien Van Olmen

**Affiliations:** 1 Department of Public Health, Institute of Tropical Medicine, Antwerp, Belgium; 2 Department of Family Medicine and Population Health (FAMPOP), Faculty of Medicine and Health Sciences, University of Antwerp, Belgium; 3 Institute of Public Health (NIPH), Phnom Penh, Cambodia and Department of Social Science, University of Antwerp, Antwerp, Belgium; 4 Community Health Centre Ljubljana, Slovenia; 5 Centre for Population, Family & Health, Department of Social Sciences, University of Antwerp, Antwerp, Belgium; 6 Centre for Health Systems Research & Development, University of the Free State, Bloemfontein, South Africa; 7 National Institute of Public Health (NIPH), Phnom Penh, Cambodia; 8 Department of Family Medicine, Medical Faculty, University of Maribor, Slovenia; 9 Department of Family Medicine, Medical Faculty, University of Ljubljana, Slovenia; 10 Julius Global Health, Julius Center for Health Sciences and Primary Care, University Medical Center Utrecht, Utrecht University, Utrecht, Netherlands; 11 Division of Epidemiology and Biostatistics, School of Public Health, Faculty of Health Sciences, University of the Witwatersrand, Johannesburg, South Africa

**Keywords:** integrated care, scale-up, complexity, multiple case study design, qualitative research

## Abstract

**Introduction:**

The ‘*Scale-Up diaBetes and hYpertension care*’ (SCUBY) project provides evidence on scaling-up integrated care (IC) in Cambodia, Slovenia, and Belgium. This paper examines macro-level barriers and facilitators to scaling up IC in these settings.

**Methods:**

We used a multi-case study design, with each country being a case. Document review, focus groups, and stakeholder interviews were conducted. The WHO health system building blocks guided the thematic analysis. We then visualised and examined the interlinkages between barriers in each country.

**Results:**

Common challenges to scaling up IC across the three health systems relate to: governance and leadership; health workforce; inadequate health financing system; and fragmented health information systems. In Cambodia, access to non-communicable disease (NCD) services and medicine are important issues. IC scale-up is facilitated by its strong governance and public health service model in Slovenia but health workforce shortages risk progress. In Belgium, the fragmented governance system and predominant fee-for-service provider payment are important barriers. A common response to health workforce and workload challenges was task shifting: to primary care nurses in Belgium, peer supporters in Slovenia, and community health workers in Cambodia.

**Conclusions:**

Examining differences and similarities between barriers in each health system stimulated reciprocal learning. Interactions between health system barriers in specific contexts require further attention to move complex health systems forward.

## Introduction

Health systems worldwide have been struggling to respond to the increasing burden of non-communicable diseases (NCDs) and associated patient needs. NCDs account for 74% of all deaths worldwide [1]. In view of their chronic nature, individual (lifestyle) and structural (social, commercial, and political) determinants, and organisationally complex coordination, they present an important public health challenge globally [1,2].

To address the rising burden of chronic NCDs, national and international commitments have been made towards integration of care. Integrated care from a health systems perspective means “the management and delivery of health services such that people receive a continuum of health promotion, disease prevention, diagnosis, treatment, disease-management, rehabilitation and palliative care services, through the different levels and sites of care within the health system, and according to their needs throughout the life course” [3]. Integrated care (IC) thus entails, amongst others, the following components: multidisciplinary collaboration, non-episodic care, and care adapted to the person’s needs [4,5,6,7]. IC leads to better care coordination and (cost) efficiency, and improves quality of care and patient outcomes by linking services along the care continuum [4,8,9]. However, barriers to IC exist at individual, organisational, and political/system levels. These barriers are difficult to overcome, because they are interrelated and require multi-stakeholder actions and intersectoral coordination; furthermore, there are implicit tensions due to different stakes, positions and ideations of actors [10,11]. This implies that successfully scaling up IC interventions, policy programmes or initiatives remains challenging due to the inherent complexity of effectively tackling NCDs and their key risk factors [10].

Scale-up of IC requires efforts to: (1) increase population coverage to IC, (2) diversify or expand the IC intervention package (e.g. through additional ‘content’ components, such as health education, self-management, mobile health, or improving quality), and/or (3) integrate or institutionalise IC into health system services, i.e. addressing health system structural barriers, e.g. via organisational or financial reform [12,13,14,15,16,17] (see appendix 1). The ‘Scale-Up diabetes and hYpertension care’ (SCUBY) project aims to provide evidence on the scale-up of IC for type 2 diabetes (T2D) and hypertension (HT) in dissimilar types of health systems through the assessment of the current status of IC implementation and through the development and evaluation of scale-up roadmap strategies that can be adapted for use in different contexts [15]. As a multi-case study, three countries were selected for evaluation of the status of IC scale-up and co-creation of scale-up roadmaps: a developing health system in a lower-middle-income country (LMIC; Cambodia); a centrally-steered health system in a high-income country (HIC; Slovenia); and a publicly-funded, healthcare system with autonomous healthcare providers in a HIC (Belgium) [15]. These three countries were chosen based on their health system characteristics as well as current focus on scale-up strategies. In a first exploratory phase, a detailed understanding of the current status of scaling up, its barriers and facilitators, and progress being made at the country level is required.

### Aim of this paper

Recently published studies show that barriers and facilitators to scaling up IC largely depend on the macro-level (policy/political or structural) context of a country, e.g. cultural inertia, type of health system, laws and regulations [18,19]. However, studies in health policy and systems research mainly focus on clinical or organisational strategies to improve health outcomes, while macro-level strategies remain underreported [19,20,21]. Hence, more knowledge is needed about what hinders and promotes scaling up IC at various levels and importantly, the connection between barriers, beyond the clinical (micro) and organisational (meso) level [22,23]. This paper addresses the above-mentioned gap and aims to: (1) identify and compare macro-level barriers and facilitators to scaling up IC in different health system contexts being Cambodia, Slovenia, and Belgium; and (2) visualise interactions between macro-level barriers and barriers at other levels. This way, lessons can be drawn on the influence and pathways of different barriers to scaling up IC in different contexts.

## Research methods

### Design

We used a multi-case study design, in which each country is a case [24]. We identified and compared barriers and facilitators to scaling up IC in these countries to learn from their context-specific differences and similarities, adopting a reciprocal learning approach [25].

#### Contexts

Since its destruction during the Khmer Rouge Regime (1975–79), the Cambodian health system has been reconstructed, with relatively substantial international assistance since 1993 [26]. Current day Cambodia has a rather marginalized public healthcare system and a largely parallel sector of private care providers, a vibrant market for the public to shop around for healthcare complements [26]. The public system is developed by the government with donor support; the private sector is growing rapidly, with state regulation lagging behind, which makes a good overview of their quality and coverage difficult [15]. While there are still many people with infectious diseases, the health system is facing a growing epidemic of NCDs. The public sector is the prominent provider of preventive and in-patient services, whereas the private sector tends to dominate provision of outpatient curative consultation [27]. In the public health system, NCD care is provided mainly at NCD clinics at the provincial referral hospitals (RHs). In addition, there is a strong network of community-based health workers, some with generic functions and some trained for support for specific diseases such as T2D [28].

As a part of former Yugoslavia until its independence in 1991, like many former communist countries, Slovenia has a centralised health system. With compulsory social insurance; care is provided mainly through public (community-based health centres) and private (but publicly regulated) health facilities. The capitation provider payment mechanism is established at the primary healthcare (PHC) level and a strong gatekeeping role is performed by general practitioners (GPs) [29]. In 2011, registered nurses (RNs) were introduced into GP practices to screen for NCDs and manage patients with stable NCDs [30,31,32]. Community nurses are responsible for nurse care at patients’ homes and in reaching vulnerable patients.

The Belgian health system has been partially decentralised, following a series of devolution reforms, but is still regulated centrally (with compulsory social health insurance), via the National Institute of Health and Disability Insurance (NIHDI) [33]. Belgium has a complex system of governance, with six different governments, one federal and five at federated level, in addition to having nine ministers of health [11]. Healthcare is provided by self-employed/autonomous healthcare providers. It is based on the patient’s free choice of physician and is mainly on a fee-for-service (FFS) payment basis. This high degree of autonomy in the patient’s choice of service utilisation and choice of care provider has led to a quite fragmented system of individualised care. PHC practices are independent and differ in many aspects, such as size and support of administrative personnel. Most practices only consist of GPs (a solo or group practice), few have dieticians or nurses [33]. The implementation of IC in PHC practices is assessed as only basic [34].

More information regarding the countries’ governance, socio-economic and demographic profile, health system characteristics, and NCD-related health outcomes can be found in appendix 2.

### Data collection methods

Primary data was collected on barriers and facilitators to IC scale-up through qualitative methods during the formative phase (2019) of the SCUBY project [15]. Common thematic interview guides (see appendices 3–4) were prepared, focussing on the assessment of the current IC scale-up and barriers and facilitators in each country at three levels: micro, meso, and macro or individual, organisation, and policy, as inspired by the World Health Organisation (WHO) framework for Innovative Care for Chronic Conditions (ICCC) [35]. In-depth, semi-structured interviews (IDI) were conducted with health facility managers, policy makers, civil servants (Ministry of Health (MoH) and health insurance), representatives of professional associations, non-governmental organisations (NGOs), implementers, academics, and patient platforms in the three countries. Additionally, focus group discussions (FGD) were organised with patients, health workers, and community-based actors in Slovenia and Cambodia to enrich the interview data. A full overview of focus group and interview participants per country can be found in appendix 5.

IDIs and FGDs were conducted in person by a minimum of two researchers. They lasted 50–90 minutes and were audio recorded. Written informed consent was obtained. Data was collected until saturation.

We also reviewed relevant literature and documents for each country to supplement the qualitative data. We purposively retrieved internal project reports, official reports, scientific publications, policy documents and grey literature sourced from in-country contacts [11,33,36,37,38,39,40,41,42,43,44,45,46,47]. Primary and secondary data were triangulated to corroborate findings. Table 1 outlines the details of data collection characteristics per country.

**Table 1:** Data collection methods across countries.


CAMBODIA	SLOVENIA	BELGIUM

33 IDIs (meso and macro level) 14 FGDs at 5 ODs Exploratory literature and document review	23 IDIs (meso and macro level) 15 FGDs (micro level); 7 with patients with T2D and HT, 8 with health workers, including GPs, RNs, practice and community nurses Exploratory literature and document review	28 IDIs (meso and macro level stakeholders, selected from one federated (Flemish) and federal level) Exploratory literature and document review


Note: FGD = focus group discussion, GP = General Practitioner, HT = Hypertension, IDI = in-depth interview, OD = Operational district RN = Registered Nurse, T2D = Type 2 Diabetes.

### Data analysis

Data analysis was divided into two phases: the per-country analysis phase and the cross-country analysis phase.

The ICCC Framework levels (macro-meso-micro) were used in the per-country analysis, distinguishing (a) patients and health professionals as the micro level, (b) community and health organisations as the meso level and (c) the underlying structures, policies and politics at the national level as the macro level [22,35,48,49,50]. The IDIs and FGDs were transcribed verbatim. NVivo software (NVivo qualitative data analysis software; QSR International Pty Ltd., https://www.qsrinternational.com/nvivo-qualitative-data-analysis-software/home) was used. At least two independent researchers carried out the analysis in their respective countries. Codes were developed in English and interpretations were discussed within the respective country teams. Aside from the common multi-level framing, three separate qualitative (inductive-deductive) thematic data analyses were conducted which resulted in three separate codebooks (one for each country).

The country analyses [11,36,44,51] offered a starting point for the cross-country analysis. The cross-country analysis phase (2022) contained several steps (see appendix 6). After the set-up of the core analysis team (MM, ČZ, SC, KD) with two supervisors (JVO, GMK) and several initial discussions, we developed a terms of reference guidebook with definitions (see appendix 7) to stimulate the use of a common language. To have a common method of analysing the three codebooks generated in the per-country analysis, a more comprehensive framework was needed. We thus retained the ICCC framework macro level and adapted the WHO’s health system building blocks [52] to include the 7^th^ building block ‘people’ [53]. Initially, we included wider contextual factors (see appendix 8), but then narrowed it down to the key health system functions and their inner complexities for the sake of scope and focus. Utilising the adapted framework, thematic analysis was conducted in a cyclical process including a deductive and inductive analysis approach. Apart from thematic analysis, visualisations were created to examine the complex interrelationships between identified barriers to IC in each country using Vensim software (Vensim PLE; Ventana Systems Inc.; https://vensim.com/).

## Results

### Identifying and comparing macro-level barriers and facilitators

Our analysis indicates that all themes of the adapted framework apply: (1) governance, (2) health service delivery, (3) health financing, (4) human resources for health (HRH)/health workforce, (5) medical supply/resources, (6) health information system (HIS), and (7) linkage between health system and community (people or demand side). Sub-themes were added inductively and deductively. Table 2 shows our key findings. In-depth case study information can be found in appendix 9 (with sub-themes) and 10 (with quotes).

**Table 2:** Identified barriers and facilitators to scale-up integrated care, based on the adapted WHO health system building blocks.


MAIN AND SUB-THEMES	CAMBODIA	SLOVENIA	BELGIUM

**1. Governance**	Limited governance for NCDs in line with limited financial commitment from government and donors and low implementation.	Strong centralised governance with strong bureaucracy, with homogeneity across country, although inefficient collaborative governance between levels and sectors for NCDs.	Fragmented, multi-level (partially decentralised) health governance necessitating a multi-stakeholder negotiation model making NCD coordination difficult.

**2. Health service delivery**	Lack of UHC with low utilisation of public sector for NCD, WHO PEN roll-out is slow. Interprofessional collaboration with nurses/midwives and CHW in PHC.	Near UHC and fairly integrated service delivery due to multi-profile teams and primary care gatekeeping.	Near UHC but variation in primary care practice organisation affecting NCD care. Collaboration in interprofessional teams largely uncommon.

**3. Health financing**	NCD low budget priority with limited financial coverage, resulting in underpayment of staff in public sector, low public coverage and high OOP.	Relatively modest total healthcare expenditure with a growing share for PHC, with low OOP. Outdated provider payment model demotivates public sector primary care providers with increasing share of private providers.	Inefficiencies in healthcare expenditure across tiers, primary care financing dominated by FFS provider payment system directly impeding IC.

**4. HRH**	HR capacity insufficient (availability, distribution, skill-mix, due to insufficient mechanisms for motivation, for instance sufficient payment, training in IC provision, leadership and management. Public workforce challenges with moonlighting and brain drain to private sector.	PHC with a relatively good skill-mix and collaboration attitude. Yet GPs are overburdened, dissatisfied, periodically threatening to strike or resign. Policies on task sharing with nurses, initiatives for further task-shifting to lay persons are being tested.	Skill-mix in PHC skewed towards GPs with increasing workload. HRH policies towards IC include new primary care models incentivising multi-professional collaboration and task-sharing, training and organisation to facilitate IC. Differentiated primary care culture across the countries hampering nation-wide roll-out.

**5. Medical supply**	Overall insufficient availability and logistic systems hampering continuous supply at public facility level, driving people towards private sector.	Well-resourced with access to all modern treatment options for patients.	Well resourced, emphasis on access to newest forms of treatment, with less focus on rationalisation of drug prescribing.

**6. HIS**	Fragmented and weak, no uniform electronic (web-based) system, no major NCD database.	Fragmented, with no interoperability of different systems. HIS registration by clinicians not prioritised hence of poor quality.	Fragmented, with no interoperability of different systems, notably that of social care with medical care. HIS registration by clinicians not prioritised hence of poor quality, also limited opportunities for population management.

**7. Link health system-community**	Focus on strengthening role of CHWs and HCWs at HCs. CHWs face challenges relating to technical and financial support, and hierarchy in relation to HCWs.	Focus on low-level care (moving care closer to the patients and their home) and task delegation (towards more emphasis on peer support).	Focus on strengthening linking PHC and social sector (difficult due to varying incentive systems in medical and social sector, e.g. FFS vs. salary) and increasing patient, social worker and local government representation and their roles in coordination of PHC.


Note: CHW = community health worker, GP = general practitioner, FFS = fee-for-service, HC = health centre, HCWs = healthcare workers, HIS = health information system, HRH = human resources for health, IC = integrated care, MoH = Ministry of Health, NCD(s) = non-communicable disease(s), OOP = Out-of-pocket expenditure, PEN = Package of Essential NCD interventions (implemented in Cambodia), PHC = primary healthcare, UHC = universal health coverage, WHO = World Health Organisation.

#### Differences between country cases

With respect to health system elements influencing the scale-up of IC, we identified the following differences between the three country cases.

In *Cambodia*, a LMIC with a developing health system, the lack of UHC is a strong impediment for IC. Low utilisation of public health services leads to fragmented – often episodic – care with very limited multidisciplinary collaboration and attention for care adaptation to the person’s needs. Structural barriers in virtually all building blocks contribute to this coverage gap, with limited resources and limited government ownership being at the roots of many.

“Government budget is not enough (…). However, external donors cannot guarantee long-term [sustainable] work.” (Representative from the MoH)

The limited NCD budget is related to a lack of political leadership and commitment to NCDs and influenced by priorities and financial resources of external partners. Two other critical bottlenecks in Cambodia are the lack of medical supply to public facilities; and a weak HIS, whereby no continuity of care (or case follow-up) is guaranteed. Despite the policy intention to roll out PEN to primary care facilities throughout the country, most NCD services (competent staff, medical drugs and supplies) are still only available at hospitals, with limited geographical coverage.

In *Slovenia*, a centrally steered small-size HIC, there is a strong PHC system in place, with multi-disciplinary teams as an essential part of the organisation, which has greatly facilitated scale-up of IC, because these teams work non-episodic, person-centred, and population-based by calling all eligible persons for annual check-up. The centralised leadership in this country facilitates a nation-wide approach.

Primary healthcare in Slovenia is based upon the vision of “comprehensive, integrated, accessible” care that is “affordable for everyone and everywhere” [42, p. 33]

Yet, signs of strain that threaten the long-term sustainability of its achievements are observed. Public dissatisfaction with the health system is growing, mainly due to long waiting times for (non-emergency) specialist care, and many dissatisfied, overworked healthcare workers (HCWs) in PHC. Furthermore, the outdated provider payment model is linked to an increase in private care provision, resulting in fragmentation of provision and increase in out-of-pocket expenditure (OOP).

In *Belgium*, a very high income country with large autonomy for HCWs, and a federated structure, the scale-up of IC has been hampered by the complicated governance structure and the variability in primary care practice. The financing and policymaking for the health system is split over federal and federated levels and healthcare organisations have a strong institutional voice in both. Both elements result in a complex power play between parties of varying interests and slow down reforms that would facilitate institutionalisation of IC:

“Integration on macro level is missing”; “there is no integrated politics” (bureaucrat)

Another context-specific barrier for Belgium is a predominantly FFS payment system (based upon consultation fees) that does not incentivise collaboration or (counter)referral of patients between HCWs. While there is a growing number of primary care practices with multidisciplinary teams and IC practices, there is a large share of monodisciplinary practices. For these HCWs and their patients, the barriers to involving other disciplines are larger due to physical, financial, organisational, and time barriers.

#### Commonalities between country cases

Common challenges in all three country cases are health workforce shortages and the development of task shifting solutions. Stakeholders in each of the three countries emphasise that task shifting facilitates scale-up of IC, either through increasing coverage or expanding the content of care in each context. The tasks shifted involve screening and self-management support amongst others, and cadres differs per country, from CHWs (Cambodia), to peer supporters/patients (Slovenia) and PHC nurses (Belgium). In all countries, stakeholders recognise the need for stronger community involvement, although the strategies are not all elaborated, nor similar. Stakeholders also note difficulties in establishing intersectoral approaches (e.g. between the healthcare and social sector or education sector). In all three case studies, the fragmentation of the HIS and limited data sharing were mentioned as crucial impediments for scaling up IC. In the absence of strong guidance by the national authorities in this matter, a myriad of different data bases has been emerging in all countries, either driven by external financing and donor needs (Cambodia) or by privatisation (Slovenia, Belgium).

### Assessing interactions between barriers at macro-level and other levels

**Figure 1:**
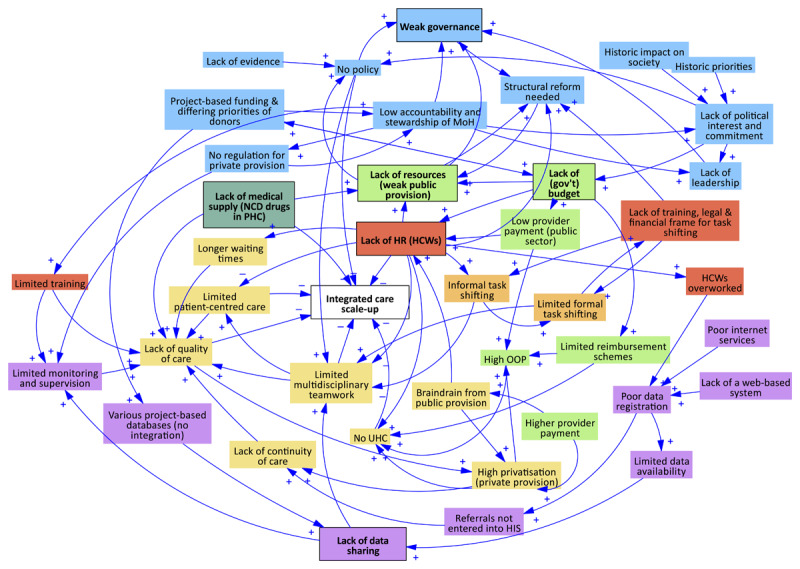
Interactions between health system barriers and facilitators to integrated care in Cambodia. Note: blue = governance; yellow = healthcare delivery; green = financing; red = HRH; dark green = medical supply; purple = HIS; orange = community.

**Figure 2:**
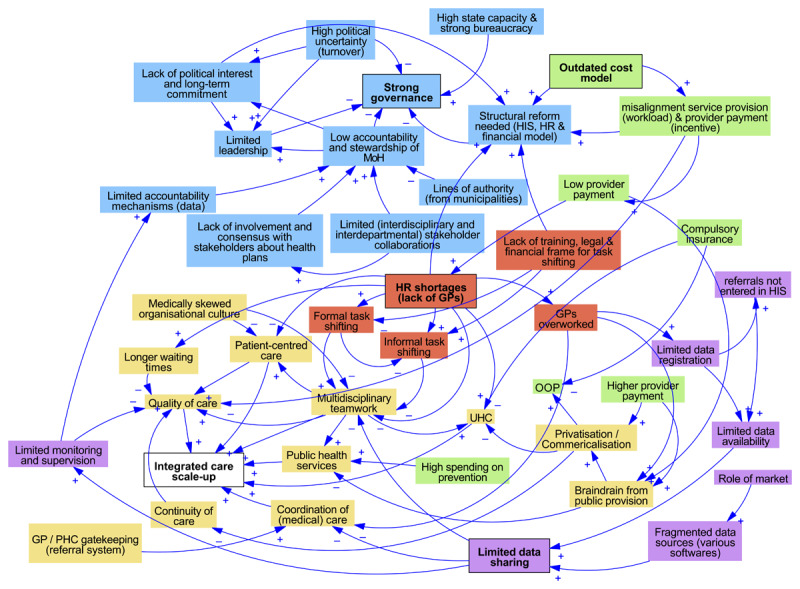
Interactions between health system barriers and facilitators to integrated care in Slovenia. Note: blue = governance; yellow = healthcare delivery; green = financing; red = HRH; dark green = medical supply; purple = HIS.

**Figure 3:**
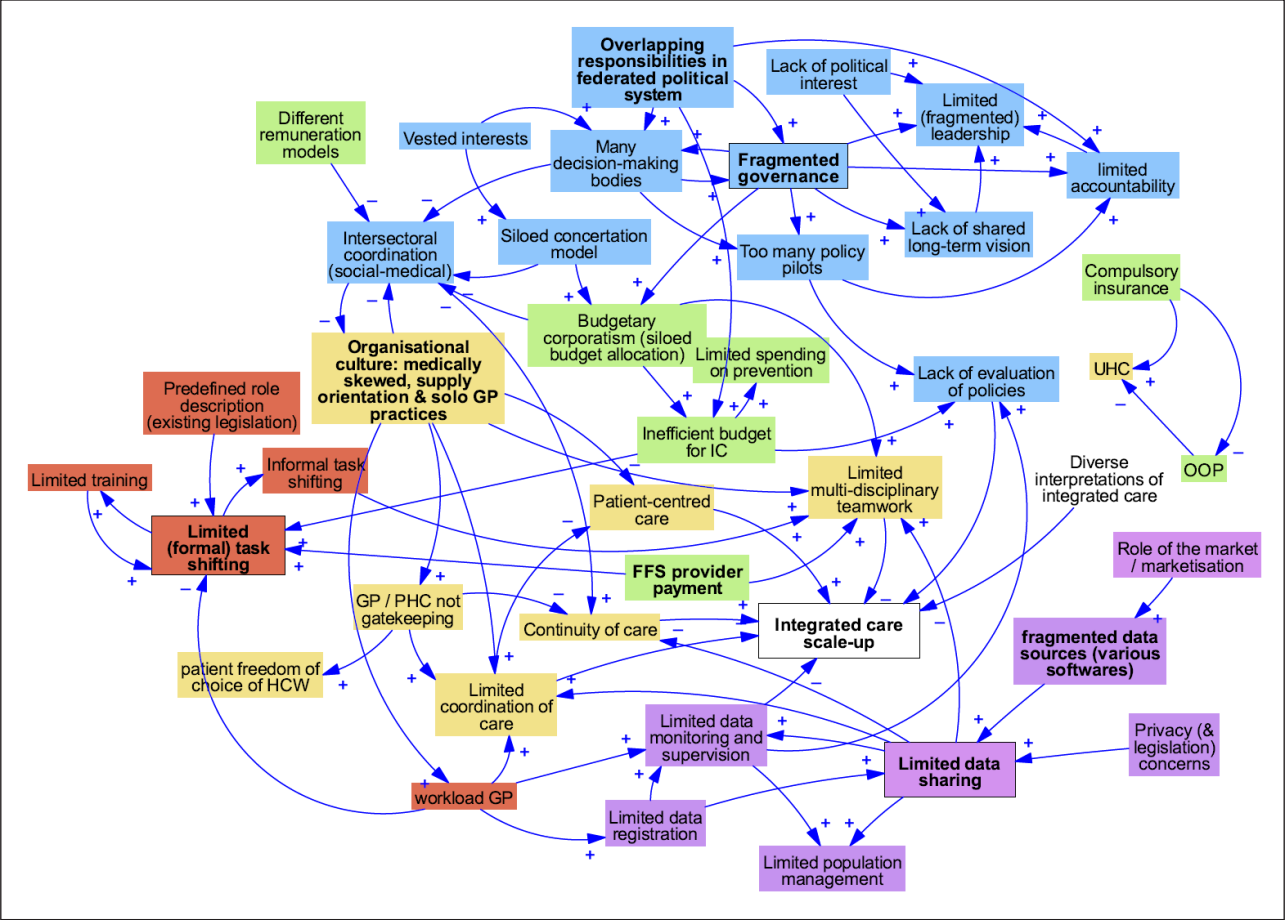
Interactions between health system barriers and facilitators to integrated care in Belgium. Note: blue = governance; yellow = healthcare delivery; green = financing; red = HRH; dark green = medical supply; purple = HIS.

Figures 1,2,3 display the interconnections between macro-level barriers and barriers at the meso- and micro-levels in each country case in this study. The arrows indicate a relationship between the elements, with the ‘+’ and ‘–’ symbol indicating a positive or negative polarity respectively.

Most notable in Cambodia (Figure 1) is the lack of (human, financial, material, medicine) resources, which impacts the quality of care and hinders scale-up of IC, in all three dimensions of coverage, content of care and institutionalisation. Since quality of care is especially lacking in the public sector and HCWs are paid better in the private sector, privatisation (through increased demand and supply) and a subsequent rise in OOP are encouraged, negatively impacting access and IC due to increased fragmentation and episodic care. These structural inequities are compounded by weak governance mechanisms, such as limited regulation for the private sector, but also limited implementation guidance and supervision for healthcare managers and providers. Privatisation also has a negative impact on HRH in the public sector: due to general staff shortages, subsequent increased workload, and low pay in the public sector more and more HCWs are enticed to move to the private sector, hence causing brain drain and moonlighting (also called ‘dual practice’ in Cambodia).

Within Cambodia’s governance system, the role of development partners is important, yet can negatively influence the accountability and stewardship by the MoH. Additionally, Cambodia faces data system and e-health challenges; poor internet services and a lack of a centralised web-based system lead to poor data registration and subsequently limited data availability, sharing, and monitoring. This hinders collaboration and coordination of care, and the monitoring of scale-up.

A defining feature in Slovenia (Figure 2) is the existence of a strong PHC system. Its centralised governance (with ‘state capability’ [42,54]) and public health approach to PHC provision are crucial to this. Well-functioning multi-profile teams have been installed within community health centres (CHCs), which positively impact the provision of public health services and IC. For example, health promotion centres were established and are connected to the CHCs. Nevertheless, there are four important health system challenges affecting the scale-up of IC, namely: GP shortages; an outdated provider payment model; HIS under-use and fragmentation; and the increasing privatisation of care provision. These are all related to each other; due to the outdated costing model, the PHC provider payment is insufficient, which causes both brain drain to the private sector as well as GP shortage. The latter leads to increased workload of GPs and subsequently, the under-use of the HIS.

At governance level, the strong governance—key for IC implementation—is challenged due to a number of structural inefficiencies (including high political turnover, leading to limited leadership; limited accountability mechanisms and stakeholder collaborations), which hinder big reforms related to the four challenges above (HRH, HIS and financial reform). Hence, if these health system and governance-related challenges are not solved, they may threaten the sustainable delivery and quality of healthcare services.

In Belgium (Figure 3), the scale-up of IC is slowed down by the fragmented and segmented decentralisation of healthcare financing and policy making that allows many pilot projects to emerge, but few institutional reforms to come through. This sustains the situation in which formal task shifting and multi-disciplinary team work are not induced. The slow progress on data sharing arrangements limits the possibility to improve continuity of care and care coordination and to monitor progress.

In sum, Belgian’s complex, fragmented, multi-level health governance itself becomes one of the biggest barriers to improving the conditions for IC and to implementation at the meso- and micro-level. The fragmentation within the policy arena leads to limited accountability and policy evaluation use and therefore, policy adaptations.

## Discussion

### Commonalities and differences in the barriers identified

We identified commonalities in our three country cases with regards to collaborative governance; health financing; HIS; and HRH. As our cross-case analysis illustrates and is confirmed by other cases, the nature of barriers and facilitators to implementation of IC seems to be remarkably similar and consistent across HIC and low- and middle-income country (LIC/MIC) settings [55], yet the relative importance of barriers might differ. We will elaborate on the key commonalities and the implications for policy and practice.

First, in Cambodia, the quality of healthcare for chronic conditions was often more concerning. This is also true in many LICs/MICs making this a more important barrier to utilisation and effectiveness of care [49]. Stepwise implementation guides such as WHO PEN [28] can be a lever to increase coverage and quality of care for high prevalent chronic NCDs, provided there are sufficient conditions allowing provision of quality of care. Primary care in many LIC/MICs is predominantly staffed with nurses with a shorter training and a less comprehensive skill set than general practitioners.

Second, IC for NCDs are not yet as high a priority in Cambodia, as it is in Slovenia and Belgium. While the health system needs major shifts and funds to adapt to this growing burden, there are still many competing priorities and vested interests that slow down an appropriate policy response. This is the case in many LICs/MICs that are grappling still with competing budgetary needs to put more means into prevention, control and management of NCDs [55].

Third, in HICs, the case studies Slovenia and Belgium, the increasing workload due to increasing number of patients with complex problems clearly identifies the need to change current ways of working towards more collaboration and interdisciplinary care and to involve communities. However, the highly professionalised (and specialist) healthcare context and subsequent vested interests are a constraint to task shifting and community involvement. LMICs can provide valuable lessons and insights on how to decrease professional barriers and boundaries [56].

### Interacting barriers

As Pirrone and colleagues noted:

“prioritizing some factors is not appropriate and that researchers should try to understand them all together, and the interplay of different levels” [23, p. 5].

Our complex systems visualisations provided insight into the identified barriers and their interactions specific for each country case. Expectedly, there were *differences in these interactions* due to the varying contexts of the three country cases.

Previous research on the complexity of insufficient resources [49] may help explain the findings in Cambodia, where it is mainly the inadequacies of the public PHC sector that hamper IC scale-up and quality of care. Consequently, many people go to the private sector, where OOP is higher, negatively affecting access to care and IC itself. Scaling up IC requires investing into quality of care and access to all elements of IC [3,15]. If this chain is only partly accessible or dysfunctional, then people risk receiving fragmented care. In Cambodia, for instance, this is apparent when people get medication at the private sector, but are not educated about their disease/condition. In Belgium and Slovenia, the care continuum could be further improved as well; e.g. via an appropriate mix of provider payment mechanisms. While access and coverage are not major concerns for the population at large in these HICs given the compulsory health insurance, inequities in access exist with people in vulnerable situations, such as the elderly, people with multimorbidities and people from lower socio-economic backgrounds [15,57]. The lack of collaboration between the health and social sector is a special concern for vulnerable groups, since many people in these groups have combined healthcare and social care needs. In Slovenia, the sustainability of the PHC system as a strong foundation of IC scale-up is at risk due to its HRH shortages, outdated financial incentive model, and HIS challenges [42,58]. In Belgium, willingness to change is needed to build a political and organisational culture that fosters IC [44].

There are also interesting *similarities in interactions between barriers*:

1. In all three countries, there are *HRH shortages within PHC* and consequently, interviewed stakeholders highlighted the *opportunities for task shifting*, which could alleviate the burden placed on HCWs. This HRH shortage is not common only to the three country cases, but is rather a universal occurrence globally [59,60,61]. In both HICs and LICs/MICs, redistribution of tasks is being advocated and has been demonstrated to be effective [62,63,64]. In the three country cases, we found that ‘informal’ or ad hoc task shifting is in place (e.g., peer support groups in Slovenia, CHWs in Cambodia and some primary care nurses in Belgium), while formalisation through accredited training, recognition, and a legal and financial frame (with clear role descriptions and incentive system, respectively) is lagging behind. For instance, CHWs in Cambodia often work as volunteers and are unpaid. In the case of Belgium, medical regulations currently prevent task shifting within PHC practices. In Slovenia, task shifting is quite formalised for registered and community nurses, but not yet for peer supporters. In some cases, task shifting is regarded as a temporary stop-gap rather than a solution [65,66]. The difficulty in formalising task shifting underlines the importance of clarifying roles, but also illustrates the dynamic and political nature of health systems, because roles and functions keep changing, and require continuous adaptations and negotiations between different actors in the system.
2. The HRH shortage redounds to the *HIS*: when HCWs are overworked, data registration at practice level is limited and, consequently, data availability, monitoring and even the potential of data sharing is weakened. Hence, the HIS is a major impediment in our country cases, but potential game changer to scale-up IC; electronic health records that allow easy and efficient registration of all relevant parameters related to chronic disease management will facilitate follow-up, clinical decision-making, continuity of care across time and between different HCWs. If extraction of data is possible, these systems can further facilitate monitoring of chronic disease parameters, assessment of quality of care and population management.
3. In both Cambodia and Slovenia, fragmentation of service provision and an increased OOP due to *privatisation* is a cause for concern. Yet, while privatisation is an increasing trend in Slovenia, the extent to which private services are offered (22% in PHC in Slovenia in 2019 [45]) and are important for NCD delivery is much lower than Cambodia, where almost 60% of people with chronic diseases are diagnosed and treated in private facilities [46]. Moreover, a strict regulation of both quality and remuneration via health insurance in Slovenia partially safeguards against the excesses of private services hampering chronic care.

Scaling up IC remains complex, meaning that actions with a focus on of the three dimensions (coverage, diversification, and institutionalisation), are intrinsically linked with and have ripple effects on the other dimensions [67]. This paper focuses on the macro-level context and processes related to IC scale-up, which are often ‘institutionalised’ health system characteristics. It thereby starts from analysing the institutionalisation dimension, and its linkage with and effect on the dimension coverage of IC and the content and quality of the IC package. The interdependency of the three scale-up dimensions coverage, content and institutionalisation is an important topic for future scale-up research.

### Strengths and limitations

Our study has a number of *content-related (theoretical)* and *operational* strengths and limitations.

In relation to *content* and scope, our analysis could be strengthened by further considering contextual (e.g. political, economic, socio-cultural, technological, environmental, and legal) factors beyond the health system [23]. However, this was out of scope for this work.

Several authors have critiqued the use of health system building blocks, arguing that it is not suitable for analysing dynamic, complex and inter-linked systems impacts [58,68]. For this reason, our thematic analysis was expanded to incorporate the missing ‘demand’ component (theme 7). We also wanted to add scope for interactions between components offering a more overarching, holistic health systems viewpoint. This was done by complementing the thematic analysis with health system visualisations which capture some of the complex relations between barriers to IC. The same authors [58,68] also emphasised the benefits of the health system building blocks framework, stating it is valuable because of its simplicity and ability to provide a common language for researchers, which were important reasons we opted for this framework.

More emphasis was placed on barriers than facilitators in the analysis. However, facilitators also open up opportunities for reciprocal learning from other contexts. We therefore suggest that more research on facilitating factors could be conducted, e.g.: to understand what constitutes strong public health services within PHC in Slovenia; how and why nurses and/or CHWs work well (or not) in PHC teams and under which circumstances; or examining contextual conditions and stakeholders dynamics of task shifting.

An *operational* constraint of multi-country research is to reach and maintain a common understanding of the concepts. For instance, the boundaries between micro, meso, and macro levels (of the ICCC framework) were difficult to establish. Even within the health system building blocks framework, such distinctions are not clear-cut. Specifically, the ‘healthcare delivery’ (theme 2) and ‘health system-community linkage’ (theme 7) building blocks act more at organisational (meso) level, but were beneficial to explore when looking at interactions between various levels.

While relationships between barriers are context-specific and our visualisations based on our qualitative evidence, in reality, most of these relationships have multiple causal roots, all of which could not be drawn up. Still, showcasing complex interactions may provide directions to policymakers on how to improve IC implementation and scale-up in a sustainable way [13]. Future in-depth case studies taking a complex systems approach could help to further disentangle important relationships between barriers and generate evidence supporting system-wide interventions tackling the many complex interactions that constrain IC implementation and scale-up.

## Conclusion

Our qualitative multi-case study was undertaken to provide a better understanding of health system barriers and, to a lesser extent, facilitators to the scale-up of IC in Cambodia, Slovenia, and Belgium.

Examining the differences and similarities between barriers and facilitators in the different health systems stimulated reciprocal learning. Specifically with regards to task shifting, Slovenia can learn from Cambodia on community health and peer support workers and Belgium from Slovenia on integrating nurses into primary care.

Our findings enrich information needed for policies and strategies to promote and scale-up of IC in Cambodia, Slovenia, and Belgium, but can also be useful in other settings with similar contexts.

## Additional File

The additional file for this article can be found as follows:

10.5334/ijic.7650.s1 Appendices. Appendix 1 to 9.

## Figures and Tables

**Table:** 

BoD	Burden of disease
CHC	Community health centre
CHW	Community health worker
CLD	Causal loop diagram
FFS	Fee-for-service
GDP	Gross Domestic Product
GP	General practitioner
HC	Health Centre
HCW	Healthcare worker
HEC	health education centre
HIS	Health information system
HRH	Human resources for health
HT	Hypertension
IC	Integrated care
ICCC	Innovative care for chronic conditions
MoH	Ministry of Health
NCD	Non-communicable disease
NIHDI	National Institute of Health and Disability Insurance
NGO	Non-governmental organisations
OD	Operational district
OOP	Out-of-pocket expenditure
PHC	Primary Healthcare
PCZ	Primary Care Zone
PEN	Package of essential NCD interventions
PESTEL	Political-Economic-Socio-cultural-Technological-Ecological-Legal (factors)
PHC	Primary healthcare
RH	Referral hospital
RN	Registered nurse
SCUBY	‘SCale-Up diaBetes and hYpertension care’ project (website: scuby.eu)
T2D	Type 2 Diabetes
THE	Total health expenditure
UHC	Universal health coverage
WHO	World Health Organization
