# Supplementary material for: Examining Macro-Level Barriers and Facilitators to Scaling Up Integrated Care from a Complexity Perspective: A Multi-Case Study of Cambodia, Slovenia, and Belgium
Source: Int J Integr Care. 2024 Nov 12;24(4):8. doi: 10.5334/ijic.7650 (PMC11568809; doi:10.5334/ijic.7650)
Supplement: Appendices. — Appendix 1 to 9. [file ijic-24-4-7650-s1.zip › ijic-7650_martens-s1/6501ba377ebc0.docx]

# Appendices/supplementary material

## Appendix 1. Three-dimensional scale-up framework


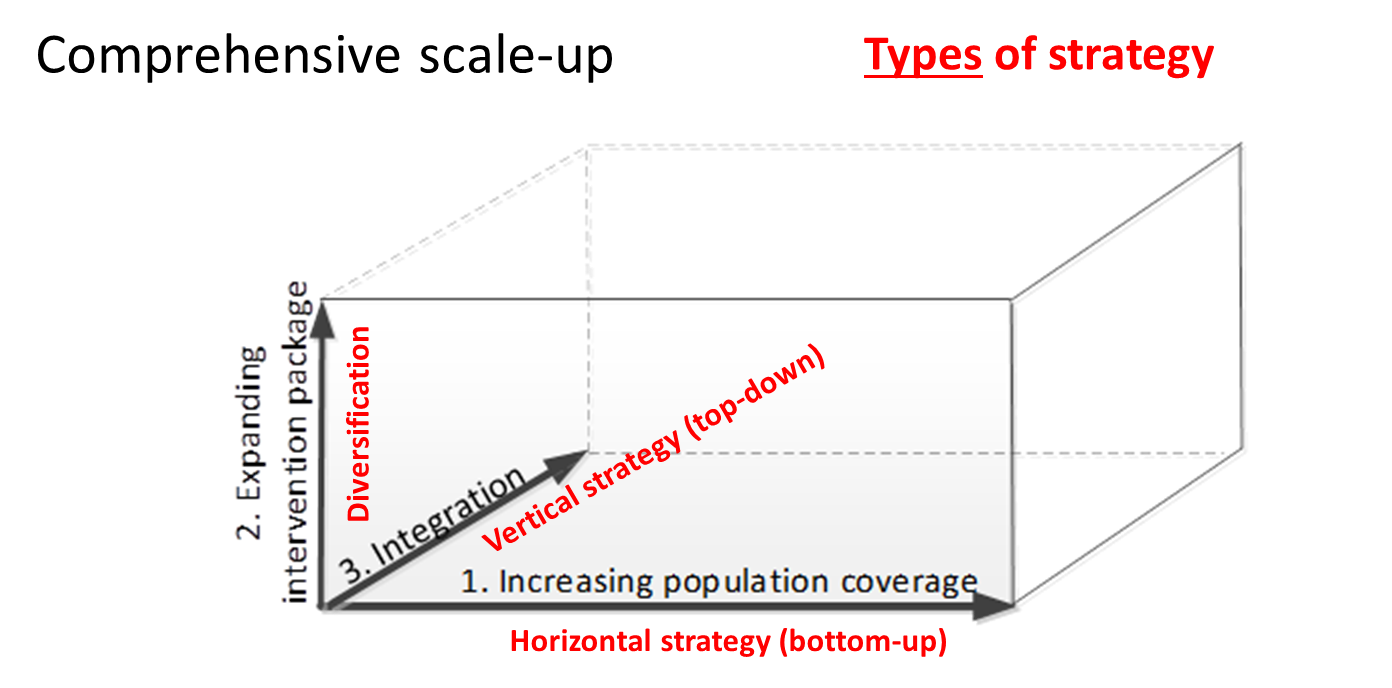


Note: Adapted from van Olmen J, Menon S, Poplas Susic A, Ir P, Klipstein-Grobusch K, Wouters E, et al. Scale-up integrated care for diabetes and hypertension in Cambodia, Slovenia and Belgium (SCUBY): a study design for a quasi-experimental multiple case study. Glob Health Action. 2020;13(1):1824382. Epub 2020/12/30. DOI: 10.1080/16549716.2020.1824382
