# Supplementary material for: Examining Macro-Level Barriers and Facilitators to Scaling Up Integrated Care from a Complexity Perspective: A Multi-Case Study of Cambodia, Slovenia, and Belgium
Source: Int J Integr Care. 2024 Nov 12;24(4):8. doi: 10.5334/ijic.7650 (PMC11568809; doi:10.5334/ijic.7650)
Supplement: Appendices. — Appendix 1 to 9. [file ijic-24-4-7650-s1.zip › ijic-7650_martens-s1/6501ba50112df.docx]

## Appendix 2. Overview of socio-economic situation, health system characteristics and NCD profile in the three study settings

|  | **Cambodia** | **Slovenia** | **Belgium** |
| --- | --- | --- | --- |
| **Governance** | | | |
| - Government type | Sovereign state, a unitary dominant-party parliamentary elective constitutional monarchy | Unitary parliamentary (post-socialist) democratic republic | Federal parliamentary democracy under a constitutional monarchy |
| - Country wealth | Lower-middle income | High income | High income |
| - Regional organisation | 25 provinces (first-level administrative division), subdivided into 159 districts and 26 municipalities (second-level administrative divisions) | 62 administrative districts or units as subdivisions of national government administration and 212 municipalities (including 12 urban municipalities) as sole bodies of local autonomy, with no intermediate level between municipalities and the Republic of Slovenia [[1](#_ENREF_1)] | There are three levels of power in Belgium comprising the Federal authorities, Federated entities (three regions based on territory and three communities based on language – Dutch, French, and German) and local authorities (provinces and municipalities) [[2](#_ENREF_2)] |
| **Socio-economic profile** | | | |
| - GDP/Capita in 2021 [[3](#_ENREF_3)] | 1,661.7 USD | 29,200.8 USD | 51,767.8 USD |
| - GDP growth rate in 2021 [[4](#_ENREF_4)] | 3.0% | 8.1% | 6.2% |
| - Income inequality (Gini index) | 30.8 (2012) [[5](#_ENREF_5)] | 24.4 (2019) [[6](#_ENREF_6)] | 27.2 (2019) [[6](#_ENREF_6)] |
| - Poverty rate [[7](#_ENREF_7)] | 17.7% (2012) | 12.4% (2019) | 14.1% (2019) |
| **Health system characteristics** | | | |
| - Health system organisation | Pluralistic–less regulated public health system and large little regulated private sector (important role of NGOs) | Centralised [[1](#_ENREF_1)] public health system with growing regulated private sector | Federal system, with partially decentralised (fragmented) responsibilities, mainly private providers |
| - Organisation | 102 ODs (each serving 100,000–200,000 people), 9 national hospitals, 25 provincial RHs, 89 district RHs (approx. one for each OD), and 1,205 health centres (covering 8,000–12,000 people) [[8](#_ENREF_8), [9](#_ENREF_9)] | Inpatient hospital care is provided by 30—mostly public—hospitals, while primary care is mostly provided by a network of 63 multidisciplinary community-based primary healthcare centres, owned and run by the municipalities [[1](#_ENREF_1), [10](#_ENREF_10)], in addition to private facilities contracted by the Health Insurance Institute | 104 acute care hospitals, 60 psychiatric hospitals, 9 specialised or geriatric hospitals [[2](#_ENREF_2)], 60 primary care zones in Brussels and Flanders for coordination, many solo GP practices (61% in 2018); 39% in group practices with other GPs; and 6% within capitation system [[11](#_ENREF_11)] |
| - Health financing system | State and large private system | Societal (social insurance) | Societal (social insurance) |
| - Healthcare provision | Mixed provider system (large private system, with little regulation and enforcement especially at primary care level) | Mixed system of public-private providers, regulation of package and tariffs  Strong public primary care system | Mixed system of public and private providers (mainly private providers, regulation of package and tariffs with some degree of freedom)  Hospital-oriented  High supply & choice-oriented public system |
| - Primary care purchasing and payment | Public providers paid fixed salary, private providers FFS | Mixed payment of capitation/FFS for all | Providers mainly opt for FFS, but there is also a capitation-based remuneration system |
| - Embedding of primary care in community network | Variable: community-based workers throughout the country but links with primary care variable | Strong with a community based nurse in primary care practice | Medium, recent reforms aim for population based IC |
| - Health expenditure   (USD/capita) in 2019 [[12](#_ENREF_12)] | 316.05 USD | 3,629.01 USD | 5,846.90 USD |
| - Health expenditure   (% of GDP) in 2019 [[13](#_ENREF_13)] | 6.99% | 8.52% | 10.66% |
| - Number of physicians per 1,000 inhabitants [[14](#_ENREF_14)] | 0.2 (2014) | 3.2 (2018) | 6.0 (2019) |
| - Number of primary care physicians per 1,000 inhabitants [[15](#_ENREF_15)] | n.d. | 1.19 (2020) | 0.62 (2020) |
| - Number of nurses per 1,000 inhabitants [[16](#_ENREF_16)] | 1.0 (2019) | 10.2 (2018) | 11.8 (2015) |
| **Demographics** | | | |
| - Total population [[17](#_ENREF_17)] | 16,487,000 | 2,079,000 | 11,539,000 |
| - Population older than 65 (% of total population) [[18](#_ENREF_18)] | 5% (2021) | 21% (2021) | 20% (2021) |
| - Life expectancy at birth, total (years) [[19](#_ENREF_19)] | 70 (2020) | 81 (2020) | 81 (2020) |
| - Probability of premature mortality from NCDs | 23% | 11% | 11% |
| - Percentage of deaths from NCDs [[17](#_ENREF_17)] | 68% | 90% | 86% |

*Note: The following abbreviations are used in the table: GDP = Gross Domestic Product, GP = General practitioner, IC = Integrated care, NCD = Non-communicable disease, n.d. = no data, NGO = Non-governmental organisation, OD = Operational District, RH = Referral hospital, USD = United States Dollar*

**References**

1. European Observatory on Health Systems and Policies. Slovenia: health system review 2021. 2021 [1 April 2023]; Available from: https://eurohealthobservatory.who.int/publications/i/slovenia-health-system-review-2021.

2. European Observatory on Health Systems and Policies. Belgium: health system summary. 2021 [1 April 2023]; Available from: https://eurohealthobservatory.who.int/publications/i/belgium-health-system-summary.

3. World Bank. GDP per capita (current US$) | Data. World Bank Open Data; 2022 [1 April 2023]; Available from: https://data.worldbank.org/indicator/NY.GDP.PCAP.CD.

4. World Bank. GDP growth (annual %) | Data. World Bank Open Data; 2022 [1 April 2023]; Available from: https://data.worldbank.org/indicator/NY.GDP.MKTP.KD.ZG.

5. World Economics. Cambodia’s Gini Year. 2021 [1 April 2023]; Available from: https://[www.worldeconomics.com/Inequality/Gini-Year/Cambodia.aspx](http://www.worldeconomics.com/Inequality/Gini-Year/Cambodia.aspx).

6. World Bank. Gini index | Data. World Bank Open Data; 2022 [1 April 2023]; Available from: https://data.worldbank.org/indicator/SI.POV.GINI?name_desc=false.

7. World Bank. Poverty headcount ratio at national poverty lines (% of population) | Data. World Bank Open Data; 2022 [1 April 2023]; Available from: https://data.worldbank.org/indicator/SI.POV.NAHC?name_desc=false.

8. Ministry of Health - Cambodia. National standard operating procedure for diabetes and hypertension management in primary care 2019. Phnom Penh: MoH; 2019 [1 April 2023]; Available from: https://niph.org.kh/niph/uploads/library/pdf/GL240_PEN-SOP_DM_and_HBP_1_APR_2019-EN.pdf.

9. Ministry of Health - Department of Planning and Health Information - Cambodia. Annual Health Sector Progress Report 2018 and Way Forwards for 2019. 2019 [25 April 2023]; Available from: <http://moh.gov.kh/content/uploads/2017/05/2019_MoH-Final01-Low.pdf>.

10. European Observatory on Health Systems and Policies. Slovenia: health system summary. 2021 [1 April 2023]; Available from: https://eurohealthobservatory.who.int/publications/i/slovenia-health-system-summary.

11. Detollenaere J, Christiaens W, Dossche D, Camberlin C, Lefèvre M, Devriese S. Barriers and facilitators for eHealth adoption by general practitioners in Belgium. Health Services Research (HSR) - KCE Reports 337. Brussels. : Belgian Health Care Knowledge Centre (KCE); 2020 [1 April 2023]; Available from: https://kce.fgov.be/en/publications/an-eq-5d-5l-value-set-for-belgium-how-to-value-health-related-quality-of-life/barriers-and-facilitators-for-ehealth-adoption-by-general-practitioners-in-belgium.

12. World Bank. Current health expenditure per capita, PPP (current international $) | Data. World Bank Open Data; 2022 [1 April 2023]; Available from: https://data.worldbank.org/indicator/SH.XPD.CHEX.PP.CD.

13. World Bank. Current health expenditure (% of GDP) | Data. World Bank Open Data; 2022 [cited 1 April 2023]; Available from: https://data.worldbank.org/indicator/SH.XPD.CHEX.GD.ZS.

14. World Bank. Physicians (per 1,000 people) | Data. World Bank Open Data; 2022 [cited 1 April 2023]; Available from: https://data.worldbank.org/indicator/SH.MED.PHYS.ZS.

15. OECD. Health Care Resources : Physicians by categories. OECD.Stat.; 2023 [1 April 2023]; Available from: https://stats.oecd.org/Index.aspx?QueryId=30173.

16. World Bank. Nurses and midwives (per 1,000 people) | Data. World Bank Open Data; 2022 [cited 1 April 2023]; Available from: https://data.worldbank.org/indicator/SH.MED.NUMW.P3.

17. World Health Organization. Noncommunicable Diseases Progress Monitor 2022. Geneva: World Health Organization; 2022 [1 April 2023]; Available from: https://[www.who.int/publications/i/item/9789240047761](http://www.who.int/publications/i/item/9789240047761).

18. World Bank. Population ages 65 and above (% of total population) | Data. World Bank Open Data; 2022 [cited 1 April 2023]; Available from: https://data.worldbank.org/indicator/SP.POP.65UP.TO.ZS.

19. World Bank. Life expectancy at birth, total (years) | Data. World Bank Open Data; 2022 [cited 1 April 2023]; Available from: https://data.worldbank.org/indicator/SP.DYN.LE00.IN?name_desc=false.
