# Supplementary material for: Examining Macro-Level Barriers and Facilitators to Scaling Up Integrated Care from a Complexity Perspective: A Multi-Case Study of Cambodia, Slovenia, and Belgium
Source: Int J Integr Care. 2024 Nov 12;24(4):8. doi: 10.5334/ijic.7650 (PMC11568809; doi:10.5334/ijic.7650)
Supplement: Appendices. — Appendix 1 to 9. [file ijic-24-4-7650-s1.zip › ijic-7650_martens-s1/6501ba6cf0bac.docx]

## Appendix 3. Thematic interview guide for in-depth interviews

| **0. Introduction** |
| --- |
| **Introduction of the researcher(s)**   - Thank - Name & function of researcher - Share researcher’s contact details (e.g. business card) - In case of 2 researchers: one asking questions (mainly) and one/both taking notes   **Introduction of SCUBY**   - SCUBY is a 4-year research project on the scaling up of integrated care for diabetes and hypertension through co-creation with key stakeholders, such as you ... We are studying the scaling up of integrated care for diabetes and hypertension, with special attention to vulnerable people. - The aim of this research is to engage with key stakeholders at all levels, to identify opportunities and barriers to integrated chronic care at the macro level, to implement and support best practices on a larger scale. - Focus on integrated chronic care, diabetes, hypertension & vulnerable groups (key terms)   **Explain purpose and the intent of the interview:**   - Aim of interview: gaining insight into your position in relation to integrated care and current policy initiatives and mapping of the macro context - Why interviewee was selected - Duration of interview (max 60 min) - Ensure anonymity and confidentiality - Questions?   **Informed consent**   - Ask (to sign) the informed consent and permission to record the interview   **Outline of interview (topics to cover)**   - Integrated chronic care - Policy & practice - Stakeholders - The financing system - Remarks and conclusion |
| 1. **Warm up questions** |
| **On the position of the representative within his/her organization:**  Q#1.1 - Can you briefly describe your position and role within your organization?  **On the organization:**  Q#1.2 - Can you shortly describe the main goals and main activities of your organization? |
| 1. **Integrated chronic care** |
| **General understanding of integrated chronic care**  Q#2.1 - What is your **understanding** of integrated chronic care? (what are key elements in your experience?) [knowledge of the policy]  Q#2.2 - How is integrated care relevant to diabetes and cardiovascular care? [knowledge of the policy]  Q#2.3 - Which of these categories best describes **your opinion** on integrated care (as defined by [policy]*) for diabetes and hypertension patients? (Read the options and circle the answer given.) [position - self]  a) I strongly support it (S)  b) I somewhat support it (MS)  c) I do not support nor oppose it (N)  d) I somewhat oppose it (MO)  e) I strongly oppose it (O)  Q#2.4 - What kind of **potential benefits do you see for your organisation** of integrated care for diabetes and hypertension patients (as defined by … [policy]*)? [interest]  Q#2.5 - What do you think that are **potential disadvantages/difficulties/threats for your organisation** of integrated care (as defined by … [policy]*)? [interest]  Q#2.6 - How do you see **your role** in the current state of implementation of the *[ICP]***? [power]  Q#2.7 - How do think that the current system can move towards integrated chronic care in our country? Which **strategies** can be used? Would you / your organisation be interested in taking the initiative in this move? [leadership]  **Define ICP** (5 ICP components from SCUBY and in practice at context/country level)  Notes:  * Integrated care is defined by means of the relevant country policy(ies):  - Care trajectories, federal bottom up initiatives and regional health reform in Belgium (three different policies presented via a policy map)  - CPC / CPC plus in Slovenia  - PEN and Peer Educator Network in Cambodia  **ICP; i.e. the existing, current practices at country level |
| 1. **Policy and practice** |
| **POLICIES** [power: ability to mobilize resources]  Q#3.1 - Which/where are according to you the **barriers and challenges** for better care integration?   - - In the political system?   - In the decision making processes?   - According to the division of responsibilities at different levels?   - In the healthcare financing system?   - Information technology?   - Available resources?   - Available competences?     - Local managers/governance     - Care providers     - Patients   - Other aspects of healthcare system?   **RESOURCES**  Q#3.2 - What are the resources you have available (human, financial, technological, political, and other – so money, people, devices…)? [resources]  Q#3.3 - How quickly can these resources be mobilised? What is the main hurdle and what can you do to optimize care? [resources]  **BEST PRACTICES**  Q#3.4 - Are there any **examples of policies/projects** that were – in your view – successful in improving ICP for diabetes and hypertension patients? What are the **‘best practices’**? **Why** were they successful? **What can we learn** from these examples? Are those projects still alive or why do you think that they didn’t manage to stay part of the system? [resources] |
| 1. **Stakeholders** |
| **STAKEHOLDERS**  We would now like to ask you a few specific questions about your opinion regarding others'  opinions of the implementation of ICP.  Q#4.1 - What are **the** **key stakeholders** [to work with on ICP] (in your view / according to you)? Whose role is **central** in the achievement of IC? Who should be **in charge** of which reforms? [alliance/leadership]  Q#4.2 - How would you describe your **collaboration** with or relation to the key stakeholders? How they interact with other caregivers and social organisations? Who do you work closely with? [alliance]  Q#4.3 - What other organizations, departments within an organization, or persons do you  think would **support** *[ICP]*? (Probe for MOH and non-MOH stakeholders) [position - others]  Q#4.4 - What do you think these supporters would **gain** from *[the ICP]*? [position - others]  Q#4.5 - Which of these supporters would **take the initiative** to actively support *[the ICP]*? [leadership]  Q#4.6 - What other organizations, departments within an organization, or persons do you think would **oppose / see difficulties** *[the ICP]*? (Probe for MOH and non-MOH stakeholders) [position - others] Q#4.7 - What do you think these opponents would gain from preventing *[the ICP]*? Why would they be opposed? [position - others]  **VULNERABLE PEOPLE**  Q#4.8. - What are vulnerable populations in your view? How are they identified? [knowledge of the policy]  **Briefly explain how we define vulnerable people in SCUBY.**  Q#4.9 - How do you reach vulnerable people? [power: ability to mobilize resources]  Q#4.10 - What do you see/think that is the biggest obstacle for vulnerable groups to get the proper care? [resources] |
| 1. **The financing system** |
| Q# 5.1 What do you think about the **current health financing arrangements –what are strengths and weaknesses?** Do you have any **recommendations** to address the weaknesses? |
| 1. **Concluding remarks & conclusion** |
| **Additional comments**   - Do you have any additional remarks? - Is there something that you think we didn’t cover that is still relevant to this issue / topic? - (Is there someone else you think we should talk to, that you can identify as a key stakeholder?)   **Next steps**   - We’ll be analysing the information you and others gave us and submitting a draft report in a few months. I’ll be happy to send you a copy to review (our findings) at that time, if you are interested.   **Thank**   - **Thank you for your time**   ***Share SCUBY brochure at the end.*** |
