# Supplementary material for: Examining Macro-Level Barriers and Facilitators to Scaling Up Integrated Care from a Complexity Perspective: A Multi-Case Study of Cambodia, Slovenia, and Belgium
Source: Int J Integr Care. 2024 Nov 12;24(4):8. doi: 10.5334/ijic.7650 (PMC11568809; doi:10.5334/ijic.7650)
Supplement: Appendices. — Appendix 1 to 9. [file ijic-24-4-7650-s1.zip › ijic-7650_martens-s1/6501ba82f4061.docx]

## Appendix 4. Thematic interview guide for focus groups discussions

**The aim of the focus group discussion/Key informant interviews is:**

- to complement data collection needed to understand the organisational context.

- to understand the *different* perspectives of key stakeholders, namely frontline implementers and beneficiaries/patients, on the current process of ICP delivery and scale-up

**FOCUS GROUPS and KEY informant QUESTIONS**

Focus group discussions are planned with three groups: patients, medical doctors and other relevant health workers (nurses) or community based actors (for instance community health workers or peer educators). In a context where multidisciplinary teams are functional, we will have one FGD with all the medical doctor, other health worker and community health worker together (focus group discussion with a team: the teams can include everyone that is systematically involved in the interaction with the patient. In Slovenia, this is the group of model practice team (doctor, practice nurse, registered nurse and community nurse) and prevention team (CKZ), in Cambodia, this can include community workers and family (informal caregivers). Patients will be selected from the target population with purposive sampling (Slovenia and Belgium older that 65 and/or having multimborbidity). Exclusion criteria are: not being able to hear or talk clearly.
This tool includes the generic topic guide for the focus group discussions, which will be adapted for the different groups, contextualised and translated for each country. Group-specific questions are listed for patients and healthcare workers/teams. The main topics that are discussed in the FG are part of the ICP grid: identification, primary care treatment, health education, self-management and cooperation between providers of care, obstacles and facilitators for scaling up. General demographic questions on gender, age, multimorbidity/diseases, living environment and the type of ICP delivery model, will be collected for, to understand the variety of respondent in the group.

**GENERAL INFORMATION (Gathered through separate form, additional to informed consent)**

**PATIENTS**

**Patients**

Year of your birth ____________________

Gender male female

Working status working retired

Your disease hypertension diabetes hypertension&diabetes

Living environment Urban Rural

Environment of your doctor Urban Rural

Do you usually go to only one doctor/nurse for check-ups Yes No

Please tackle your treatment plan below

HT, HT + complications, on insulin + HT, on insulin + complications, on insulin + HT + complications, diabetic but not using insulin, diabetic but not using insulin + HT

**Teams**

Year of your birth ____________________

Gender male female

Living environment Urban Rural Environment of your work Urban Rural

**Focus group guide: outline**

| **Introduction** |
| --- |
| **Introduction of the researcher(s)**   - Thank - Name & function of researcher - In case of 2 researchers: one asking questions (mainly) and one/both taking notes   **Introduction of SCUBY**   - SCUBY is a 4-year research project on the scaling up of integrated care for diabetes and hypertension. We are studying the scaling up of integrated care for diabetes and hypertension, with special attention to vulnerable people. - The aim of this research is to engage with stakeholders at all levels, including patients and healthcare staff, to identify opportunities and barriers to integrated chronic care and to support and implement best practices on a larger scale. - Focus on integrated chronic care, diabetes, hypertension & vulnerable groups (key terms)   **Explain purpose and the intent of the interview:**   - Aim of interview: gaining insight into opinions and perceptions of patients, healthcare teams and community actors in relation to integrated care, its barriers and facilitators - Duration of focus group interview (max 60min) - Ensure confidentiality - Questions?   **Informed consent**   - Ask (to sign) the informed consent and permission to record the interview |
| **A. Questions to doctors / healthcare staff / teams** |
| **OPINION ON CURRENT PROCESS**   1. Which adaptations were made at the implementation of the integrated care package at the healthcare facility and at other organisations involved? 2. Which external organisations were involved to support implementation? 3. How were health workers, other actors and patients prepared for implementation? 4. What was the role of local and central health authorities in the implementation?   **FACILITATORS**   1. In your opinion, what is good in the **current implementation of the integrated care** for people with diabetes and hypertension (specify in each country according to CPC, PEN, etc. if needed)? 2. How are vulnerable population identified? How well does ICP reach **vulnerable groups** of people? (Slovenia/Belgium) 3. What are **important factors that facilitate** the current way of working? What are further **options for improving** management (detection, treatment, self management) of people with T2D and/or hypertension? (Slo/Bel) Especially when you think about vulnerable people? 4. How do you see the directions of **possible extension of care /scaling up** and facilitators in doing that? 5. Who are **potential people/approaches** that can be added to the existing care. 6. How do you see the **role of patients as teachers?** Of organisations in the community? Of informal caregivers?   **BARRIERS /OBSTACLES**   1. What are **your personal experiences / what kind of obstacles** (in screening, testing, treatment) have you considered in the ICP process (screening, testing, diagnosing, retained in care, follow up, self management, different levels cooperation) for (vulnerable) patients with HY/T2D at the primary level? 2. What are the **problems of people who don’t come regularly for care?** What are current actions you take? What are other options? 3. Where are the **obstacles** that would prevent expansion or change in the process?   **CARE & FINANCIAL BARRIERS (input WP3)**   1. What according to you are **major financial barriers** to appropriate healthcare for patients with DM2/HT/ DM2+HT/ DM2 with complications/ DM2+HT with complications treatment? |
| **B. Questions to patients** |
| **OPINION ON CURRENT PROCESS**   1. What is good in the existing process? 2. Where are you seeking / gaining the most **knowledge and support** / what kind of self management does it help you/do you use? 3. What view do you have on informal caregivers, patients as teachers and other informal staff? Who are **potential people/approaches** that can be added to the existing care. 4. In what way do you see the care to develop in order to give you the greatest support and to gain the most out of it?   **FACILITATORS**   1. What are your **personal experiences** with the moment you remember that you were **diagnosed with treatment?** 2. What are your **personal experiences** with the **follow up of your disease?** 3. What is good in the **existing process** (about the treatment of your disease, communication and shared decision making with healthcare workers, plan care tailored to your needs and circumstances)? 4. How do you see the **role of patients as teachers?** Of organisations in the community? Of informal caregivers?   **BARRIERS**   1. Where do you (or your family members) usually **go for care** for diabetes/hypertension? 2. Are there other people you go to for you diabetes/hypertension apart from your doctor? 3. Are there people in the environment who help you in the management of your diabetes/hypertension? 4. Are there organisations outside of the hospital that are of help in the management of your disease? 5. Do you experience problems/obstacles in getting care? Can you elaborate? 6. What do you feel is **not so good in the process?** What can be changed? 7. Where are the **obstacles** that would prevent expansion or change in the process? 8. Do you/ did you face **barriers** to access the services? Do you know other people who should come to the services, but don’t come? What are the reasons for this?   **CARE & FINANCIAL BARRIERS (input WP3)**  All countries   1. Has your **lifestyle changed** since being diagnosed? How? 2. **Describe your treatment**. (try to relate to patient profile) On which items you spend the most money for **your treatment?** Have there been any changes in your health expenditure since you were first diagnosed? What about expenditures on transportation, special food, special health activities? 3. What kind of **home equipment for self care** does your condition require**?** 4. Are there some **services available which could be useful but unaffordable or difficult to access?** Do you know people who were able to get access to such services? How do they manage? 5. Are there any **other financial views (not really barriers)** which could help you to better manage your condition? 6. Can you please describe what you do on your own for your health and better management of your disease?   Questions if country does not know the clear answer   1. What are the **services available to you** as a patient with (as per the above epidemiological profile)? 2. **How do you get access** to such services? **What are the costs** involved? 3. **Did you need to spend** to get to know your T2D and/or HT status (**detected/diagnosed**)? 4. For those who were **hospitalized: what were the costs involved?** (encourage some to discuss if more than once: did you need to use ambulatory and emergency care? ) |

| **C. Questions to community actors (Cambodia)** |
| --- |
| **FACILITATORS**  1. In your opinion, what is good in the **current implementation of the integrated care** for people with diabetes and hypertension (specify PEN or other relevant program implemented in that site)?  2. What is your **work/communication flow** with the people living in the community and Health Center Management Committee (HCMC)?  3. How well do you **reach and engage** with people in the community?  4. What are important **factors that facilitate** the current way of working?  5. What are further **options for improving** community engagement in the integrated care of people with T2D and/or hypertension?  6. How do you see the **directions of possible extension** of care /scaling up and facilitators in doing that?  7. Who are **potential people/approaches** that can be added to the existing care.  **BARRIERS**  1. What are your **personal experiences** / what kind of **obstacles** in your work as the community health worker? Working flow with community and HCMC?  2. What are the **problems of people who don’t come regularly for care**? **Don’t take action with your message/advise**? What are current actions you take? What are other options?  3. Where are the **obstacles** that would prevent expansion or change in the process?  **CARE & FINANCIAL BARRIERS (input WP3)**    1. Do you provide **specific services** to the community (especially peer educator such as offer blood glucose/ blood pressure measurement), how is the service charged? How is the **affordability and accessibility** of this services to patients in the community?  2. What according to you are **major financial barriers** to appropriate healthcare for patients with DM2/HT/ DM2+HT/ DM2 with complications/ DM2+HT with complications treatment? |
| **Concluding remarks** |
| **Additional comments**   - Do you have any additional remarks?   **Thank**   - Thank you for your time.   ***Share SCUBY brochure at the end.*** |
