# Supplementary material for: Examining Macro-Level Barriers and Facilitators to Scaling Up Integrated Care from a Complexity Perspective: A Multi-Case Study of Cambodia, Slovenia, and Belgium
Source: Int J Integr Care. 2024 Nov 12;24(4):8. doi: 10.5334/ijic.7650 (PMC11568809; doi:10.5334/ijic.7650)
Supplement: Appendices. — Appendix 1 to 9. [file ijic-24-4-7650-s1.zip › ijic-7650_martens-s1/6501ba9940260.docx]

## Appendix 5. Selection of participants for focus groups and interviews

| **Participants level** | **Slovenia** | **Belgium** | **Cambodia** |
| --- | --- | --- | --- |
| **Micro (FGs)** | FG Patients - Ljubljana (6 focus groups) | Secondary data | Patients living with T2D and/or HTN (4 groups) in  Siem Reap, Takeo, Prey Veng |
|  | FG Patients - Ravne na Koroškem |  | Healthcare staff (5 groups) in Siem Reap, Takeo, Kompong Speu, Oddormeanchey, and Prey Veng |
|  | FG Health Professionals - Ljubljana (5 focus groups) |  | Community health workers (5 groups) in  Siem Reap, Takeo, Kompong Speu, Oddormeanchey, and Prey Veng |
|  | FG Health Professionals - Lendava |  |  |
|  | FG Health Professionals - Gornja Radgona |  |  |
|  | FG Health Professionals - Ravne na Koroškem |  |  |
| **Meso (interviews)** | Community Health Centre Ljubljana (3 interviews) | Medical Association of GPs (Domus Medica, DM) | Representative of the Referral Hospital (5 interviews) |
|  | Community Health Centre Postojna | Belgian Association of Doctors Syndicates (BVAS) | Representative of the Provincial Health Department (5 interviews) |
|  | Institution for informal home care | Medical Association of GPs and Specialists (ASGB) | Representative of the Operational District (5 interviews) |
|  | (Zavod za oskrbo na domu) | General Pharmaceutical Association (APB) | Preah Kossamak Hospital |
|  | Municipality of Ljubljana (2 interviews) | Network of Hospitals (ICURO) | Sihanouk Hospital, Center of Hope |
|  | Associations of patients with chronic diseases (Društvo Za Srce) (2 interviews) | Flemish Association of Dieticians |  |
|  | Retirement home (Dom starejših občanov Tabor) | Association of Diabetes Nurses |  |
|  | Associations of patients with chronic diseases (Društvo diabetikov) | Flemish Association of Independent Nurses (VBZV) |  |
|  |  | Network of Homecare Nurses (Zorggezind) |  |
|  |  | Association of Home Nursing (WGK) |  |
|  |  | Flemish Patient Platform (VPP) |  |
|  |  | Flemish Diabetes Association (Diabetes liga) |  |
|  |  | Primary care zone (3 zones: Antwerp, Ghent, Kempenland) |  |
| **Macro (interviews)** | National Institute for Public Health (2 interviews) | Federal Public Service of Health (FOD) | Department of Preventive Medicine, Ministry of Health (3 interviews) |
|  | National Institute for Health Insurance | Flemish Cabinet | Department of Hospital Services, Ministry of Health |
|  | Ministry of Health (2 interviews) | Association of Flemish Cities and Municipalities (VVSG) | Department of Planning and Health Information, Ministry of Health |
|  | Chamber of Pharmacies |  | Department of Human Resources |
|  | Health Council at Ministry of Health | Flemish Agency of Care and Health (VAZG) | National Center for Health Promotion |
|  | Chamber of nurses, midwives and healthcare assistants of Slovenia | National Institute of Health & Disability Insurance (NIHDI) (3 interviews) | Payment Certification Agency |
|  | Medical University of Ljubljana, Department of Family Medicine (3 interviews) | Christian Health Fund (CM) | National Social Security Fund |
|  | Medical Chamber | Joint College of Sickness Funds (NIC) | MoPoTsyo Peer Education Network |
|  |  | Socialist Sickness Fund | World Health Organization, Cambodia Office |
|  |  | Federal Knowledge Centre for Healthcare (KCE) | Louvain Cooperation |
|  |  | Academia/Medical universities (2 interviews) | World Bank, Cambodia Office |
|  |  |  | GIZ (Deutsche Gesellschaft für Internationale Zusammenarbeit) |
|  |  |  | University Research Co., Ltd, Health and Social Development Project |
|  |  |  | University of Health Sciences |
