# Supplementary material for: Examining Macro-Level Barriers and Facilitators to Scaling Up Integrated Care from a Complexity Perspective: A Multi-Case Study of Cambodia, Slovenia, and Belgium
Source: Int J Integr Care. 2024 Nov 12;24(4):8. doi: 10.5334/ijic.7650 (PMC11568809; doi:10.5334/ijic.7650)
Supplement: Appendices. — Appendix 1 to 9. [file ijic-24-4-7650-s1.zip › ijic-7650_martens-s1/6501baafdbf6a.docx]

## Appendix 6. Cross-country analysis steps

| **Analysis stage** | **Description of steps taken** |
| --- | --- |
| Previous work part of country analysis phase 1 | - Step 0.1: development of initial common coding frame (in September 2019), making use of WHO’s ICCC framework levels (macro-meso-micro) [[1-5](#_ENREF_1)] - Step 0.2: country-specific analysis at different levels (macro-meso-micro) |
| Cross-country analysis phase 2 | - **Step 1**: set-up of a core analysis team (MM, ČZ, SC, KD) with two supervisors (JVO, GMK) - **Step 2**: discussion on macro-level codes cross-country in various meetings (end 2021-2022) - **Step 3**: identification of gaps in and interactions between macro-level data and performance of complementary literature review, including internal project reports, official reports, scientific publications, policy documents and grey literature sourced from in-country contacts - **Step 4**: development of terms of reference (TOR), concepts and agreed-upon definitions to stimulate the use of a common language (see appendix 6) - **Step 5**: presentation of primary and secondary data together in a framework, making use of WHO’s health system building blocks [[6](#_ENREF_6)] (following this as overall structure for main themes, whilst adding several themes inductively and from the literature [[7-10](#_ENREF_7)]) - Regular feedback (at each step) from the analysis team to the entire author team |

**References**

1. Gilson L, Alliance for Health; Policy Systems; Research - World Health Organization. Health policy and systems research: a methodology reader / edited by Lucy Gilson. Geneva: World Health Organization; 2012 [1 April 2023]; Available from: https://apps.who.int/iris/handle/10665/44803.

2. World Health Organization - Noncommunicable Diseases and Mental Health Cluster. Innovative care for chronic conditions : building blocks for actions : global report. Geneva: World Health Organization; 2002 [1 April 2023]; Available from: https://apps.who.int/iris/handle/10665/42500.

3. World Health Organization - Health Services Delivery Programme; Division of Health Systems and Public Health. Integrated care models: an overview. Geneva: World Health Organization; 2016 [1 April 2023]; Available from: https://[www.euro.who.int/__data/assets/pdf_file/0005/322475/Integrated-care-models-overview.pdf](http://www.euro.who.int/__data/assets/pdf_file/0005/322475/Integrated-care-models-overview.pdf).

4. Epping-Jordan JE, Pruitt SD, Bengoa R, Wagner EH. Improving the quality of health care for chronic conditions. Qual Saf Health Care. 2004;13(4):299-305. Epub 2004/08/04. DOI: 10.1136/qhc.13.4.299

5. Nuno R, Coleman K, Bengoa R, Sauto R. Integrated care for chronic conditions: the contribution of the ICCC Framework. Health Policy. 2012;105(1):55-64. Epub 2011/11/11. DOI: 10.1016/j.healthpol.2011.10.006

6. World Health Organization. Everybody’s business -- strengthening health systems to improve health outcomes: WHO’s framework for action. Geneva: World Health Organization; 2007 [1 April 2023]; Available from: https://apps.who.int/iris/handle/10665/43918.

7. Mounier-Jack S, Griffiths UK, Closser S, Burchett H, Marchal B. Measuring the health systems impact of disease control programmes: a critical reflection on the WHO building blocks framework. BMC Public Health. 2014;14:278. Epub 2014/03/29. DOI: 10.1186/1471-2458-14-278

8. Kielmann K, Hutchinson E, MacGregor H. Health systems performance or performing health systems? Anthropological engagement with health systems research. Soc Sci Med. 2022;300:114838. Epub 2022/03/03. DOI: 10.1016/j.socscimed.2022.114838

9. Suter E, Oelke ND, da Silva Lima MAD, Stiphout M, Janke R, Witt RR, et al. Indicators and Measurement Tools for Health Systems Integration: A Knowledge Synthesis. Int J Integr Care. 2017;17(6):4. Epub 2018/03/29. DOI: 10.5334/ijic.3931

10. Savigny Dd, Adam T, Alliance for Health Policy Systems Research, World Health Organization. Systems thinking for health systems strengthening / edited by Don de Savigny and Taghreed Adam. Geneva: World Health Organization; 2009 [1 April 2023]; Available from: https://apps.who.int/iris/handle/10665/44204.
