# Supplementary material for: Examining Macro-Level Barriers and Facilitators to Scaling Up Integrated Care from a Complexity Perspective: A Multi-Case Study of Cambodia, Slovenia, and Belgium
Source: Int J Integr Care. 2024 Nov 12;24(4):8. doi: 10.5334/ijic.7650 (PMC11568809; doi:10.5334/ijic.7650)
Supplement: Appendices. — Appendix 1 to 9. [file ijic-24-4-7650-s1.zip › ijic-7650_martens-s1/6501bac49462b.docx]

## Appendix 7. Terms of reference: agreed definitions of used concepts in analysis

| **Concept** | **Definition used in SCUBY and by the analysis team** |
| --- | --- |
| Health system | A health system consists of all the organizations, institutions, resources and people whose primary purpose is to improve health. [1] |
| **1. Governance** | Governance refers to the entirety of regulations – that is, the processes by which norms, rules and programs are monitored, enforced and adapted, as well as the structures in which they work – put forward with reference to solving a specific problem or providing a common good [2]. It involves ensuring the existence of strategic policy frameworks, effective oversight and coalition-building, provision of appropriate incentives, and attention to system design, and accountability [1, 3-7]. |
| **1.1 NCD policies and strategic plans on integrated care for chronic diseases**  *Link to:* Stewardship, oversight [8], strategic direction/vision and policy design [6, 8], strategic policy frameworks [3-7] | Stewardship offers a perspective on governance, it is an *oversight* function that is the responsibility of government and involves “setting and enforcing the rules of the game and providing *strategic direction* for all the different actors involved” [8]. It is the government, through the Ministry of Health, which remains steward of the health system [8]. |
| **1.2 Stakeholder collaborations (collaborative governance)**  *Link to:* participation and consensus orientation [6], coalition-building [3-7], networks [9-11] , collaborative governance [12, 13] | “(…) interactions with social actors such as networks, with market actors, or with other governments, but all these forms represent means of governing involving mixtures of state action with the actions of other entities” [12, 13]. Social participation requires all stakeholders in the participatory process to be able to adequately and fully exercise their roles. In order to do so, all stakeholders should be, as far as possible, on an equal footing with each other in terms of ability to have influence on the participation-based discussions [14]. |
| **1.2.1 Inter-sectoral approach**  *Link to:* intersectoral governance [15] | The extent to which solely medical approach is being abandoned; is there collaboration between medical and social sector? To what extent do other ministries, industry etc. take part in policy-making for IC? |
| **1.3 Accountability** [3-7] | “Accountability means being answerable to someone for decisions and actions. […] there are several common principles:   - Accountability in health services delivery is predicated on relationships between the ones making the decisions and those affected by them. - A set of goals or objectives must be defined including clear explanation of roles and responsibilities of each party in achieving the goals. - The accountability relationship is governed by the ability to measure and monitor if these objectives or goals are being met.” [16] - “The need for greater accountability arises both from increased funding and a growing demand to demonstrate results. Accountability is therefore an intrinsic aspect of governance that concerns the management of relationships between various stakeholders in health.” [1, 16] |
| **1.4 Leadership** | The willingness to initiate, convoke, or lead an action for or against the health reform policy [17]. |
| **1.5 Political interest and power dynamics** | The stakeholder’s interest in the policy, or the advantages and disadvantages that implementation of the policy may bring to the stakeholder or his or her organization [17]; hence the way the stakeholder is affected by the policy.  Interest is about what stakeholders have to win or lose in IC policies. This is highly contextual about the type of stakeholders in the field and the role they play.  Power entails the ability of the stakeholder to affect the implementation of the health reform policy, which determines the level of force with which the stakeholder might support or oppose the policy [17]. |
| **1.6 Political commitment** | Gore et al. [18] distinguish three types of commitment, namely: expressive commitment, institutional commitment, and budgetary commitment. “Expressed commitment refers to verbal declarations of support for an issue by high-level, influential political leaders. Institutional commitment comprises the adoption of specific policies and organisational infrastructure in support of an issue. Budgetary commitment consists of earmarked allocations of resources towards a specific issue relative to a particular benchmark. The combination of the three dimensions signals that a state has an explicit intention or policy platform to address this health area.” |
| **2. Health system delivery** | “Service provision or delivery is an immediate output of the inputs into the health system, such as the health workforce, procurement and supplies, and financing. Increased inputs should lead to improved service delivery and enhanced access to services.” [1]  Good service delivery comprises quality, access, safety and coverage [19]. |
| **2.1 Structural components** | About structures; how the delivery system is structured |
| **2.1.1 Public-private mix** | Healthcare can be provided in the public sector and/or in the private sector. Hence, there is usually a mix and this ratio, this reality, has certain implications. |
| **2.1.2 Referral system** | The structure set up to direct a patient, requiring meso-level structural coordination between care providers/organisations, e.g. from one level of care to another. Additionally, exploring the role of the *GP having a gatekeeping function (or not)*. |
| **2.1.3 Geographical coverage** [20] | Reflecting on the density of physicians/HCWs (and for example on rural vs urban differences)  Coverage: Service delivery is designed so that all people in a defined target population are covered, i.e. the sick and the healthy, all income groups and all social groups [1]. |
| **2.2 Organisational components** | About processes; how (micro-level) care is organised and characterised |
| **2.2.1 Quality of service** | “The extent to which healthcare services provided to individuals and patient populations improve desired health outcomes” – outlining safety, effectiveness, timeliness, efficiency, equity, and people-centredness of health services are integral to achieving quality [21, 22]. |
| **2.2.2 Supervision and monitoring** | Is about how to ensure accountability at organisational level. Supervision (2.2.2) can come from a higher administration level, while coordination of care (2.2.3) could come from health facility or community level. Monitoring (2.2.2) can be linked to feedback and evaluation mechanisms, but also population management (when data is aggravated). |
| **2.2.3 Coordination of care and multi-level communication/coordination between HCWs** | Micro-level coordination, around the patient, for “a functioning trans-sectoral care that interlocks all involved healthcare providers” [23]. This coordination is also requiring meso-level communication and coordination (between HCWs across disciplines and at different levels) and possibly patient referral (2.1.2).  Local area health service networks are actively coordinated, across types of provider, types of care, levels of service delivery, and for both routine and emergency preparedness. The patient’s primary care provider facilitates the route through the needed services, and works in collaboration with other levels and types of provider. Coordination also takes place with other sectors (e.g. social services) and partners (e.g. community organizations) [1]. |
| **2.2.4 Access to care (to integrated PHC)** | Services are directly and permanently accessible with no undue barriers of cost, language, culture, or geography. Health services are close to the people, with a routine point of entry to the service network at primary care level (not at the specialist or hospital level). Services may be provided in the home, the community, the workplace, or health facilities as appropriate [1]. |
| **2.2.5 Patient centeredness [20]; Person-centeredness [22]** | Services are organised around the person, not the disease or the financing. Users perceive health services to be responsive and acceptable to them. There is participation from the target population in service delivery design and assessment. People are partners in their own healthcare [1]. |
| **2.2.6 Comprehensive services across the continuum of care [20] (including role of preventive programmes)** | Capturing the adequacy and continuity of transitional care within and between acute care, primary care, and different community care services and settings/sectors [20]. |
| **2.2.7 Interprofessional teams [20]** | Looking into team effectiveness, HCWs involved in integrated health systems [20]. |
| **2.2.8 Organisational culture [20]** | Hierarchy culture, market culture, clan culture, and adhocracy culture [20]**.** |
| **3. Health financing** | Health financing refers to the “function of a health system concerned with the mobilization,  accumulation and allocation of money to cover the health needs of the people, individually and  collectively, in the health system… the purpose of health financing is to make funding available, as  well as to set the right financial incentives to providers, to ensure that all individuals have access  to effective public health and personal healthcare” [1].  “A good health financing system raises adequate funds for health, protects people from financial catastrophe, allocates resources, and purchases good and services in ways that improve quality, equity, and efficiency” [19]. |
| **3.1 Budget / funding for IC (collection of funds)** | “Raising sufficient funds for health. In low-income countries this must come from external and internal sources. Increasingly reliable external funds are needed in most countries, but more can be done to raise funds, or raise them more efficiently, domestically” [1]. |
| **3.1.1 Service user financial payment to IC (including out-of-pocket / OOP)** | The amount that is not reimbursed by insurance and thus paid by the patient, out-of-pocket. |
| **3.2 Social health protection (taxes and/or insurance scheme; risk pooling)** | “Improving financial risk protection and coverage for vulnerable groups. In most countries this requires moving away from direct out-of-pocket payments and towards a form of prepayment with risk pooling that is tax- or insurance-based” [1]. |
| **3.3 Service provider financial payment (purchasing and resources allocation)** | The way that the healthcare worker is paid, e.g. a (fixed) salary, fee-for-service, or capitation-based. |
| **4. Human resources for health / health workforce** | “The health workforce can be defined as all people engaged in actions whose primary intent is to enhance  health. These human resources include clinical staff, such as physicians, nurses, pharmacists and dentists, as well as management and support staff, i.e. those who do not deliver services directly but are essential to the performance of health systems, such as managers, ambulance drivers and accountants. […] Human resources for health include individuals working in the private and public sectors, those working full-time or parttime, those working at one job or holding jobs at two or more locations, and those who are paid or provide services on a voluntary basis. They include workers in different domains of health systems, such as curative, preventive and rehabilitative care services as well as health education, promotion and research. They may also include people with the education and training to deliver health services but who are not engaged in the national health labour market (e.g. if they are unemployed or have migrated or withdrawn from the labour force for personal reasons)” [1]. |
| **4.1 Availability / shortages** | Reflecting on the capacity and size of health workforce relative to the population, which may reflect a shortage or oversupply of certain HCWs. |
| **4.2 HRH planning / management** | “Strengthening the performance of health systems depends on more than just increasing the numbers of health workers; actions for assessing and strengthening their recruitment, distribution, retention and productivity are also important” [1]. |
| **4.3 Training** | Reflecting on skills of the workforce and vocational training |
| **4.4 Task shifting** | Task shifting involves the rational redistribution of tasks to individuals within the healthcare team with fewer qualifications that conventionally were not within their scope of work [24-26].  It is an approach to help address the shortage of healthcare workers through reallocating human resources [26]. This is about finding the best person (or machine) for the job, or in other words, task shifting is the process of matching skills to changing needs and opportunities [27]. It involves questioning what health workers do, asking if it can be done in an improved way, and implementing change [27]. |
| **5. Medical supply/resources** | “A well-functioning health system ensures equitable  access to essential medical products, vaccines and technologies of assured quality, safety, efficacy and cost-effectiveness, and their scientifically sound and cost-effective use” [1]. |
| **5.1 Medications (supply and availability)** | Are medications for NCDs supplied and available within primary healthcare? |
| **5.2 Equipment (supply and availability)** | Is equipment for NCDs supplied and available within primary healthcare? |
| **6. Health information system(s)** | “Sound and reliable information is the foundation of decision-making across all health system building blocks. It is essential for health system policy development and implementation, governance and regulation, health research, human resources development, health education and training, service delivery and financing. [...] The health information system provides the underpinnings for decision-making and has four key functions: (i) data generation, (ii) compilation, (iii) analysis and synthesis, and (iv) communication and use. [...] It is sometimes equated with monitoring and evaluation but this is too reductionist a perspective. In addition to being essential for monitoring and evaluation, the information system also serves broader objectives, such as providing an alert and early warning capability, supporting patient and health facility management, enabling planning, underpinning and stimulating research, permitting health situation and trends analyses, orienting global reporting, and reinforcing communication of health challenges to diverse users.” [1]. |
| **6.1 E-health / digital health** | The approach (specific plans, projects and initiatives) surrounding the digitalisation of health data and the development and use of digital platforms for sharing health information. |
| **6.2 Data availability, registration & monitoring** | Data collected (in registers, public health accounts etc.) usually for service monitoring, planning and feedback, e.g. using administrative, performance, clinical data |
| **6.3 Fragmentation of data sources & data sharing** | Data (e.g., administrative, performance, clinical) is tracked and shared with stakeholders in shared/joint information systems across sectors and shared patient electronic charts across continuum of care accessible to patients [20] |
| **7. Linkage health system-community (demand side)** | The original WHO health system building blocks only had 6 building blocks [1], later a ‘people’ dimension was added [6, 28]. These are collaborators and partners and this demand side perspective is often neglected [29]. |
| **7.1 Role of community organisations (formal-informal) and the community health workers (CHW)** | Who are they? What is their role? Which barrier do they aim to fill/address? |
| **7.2 Role of the patient / public** | What is their role in the healthcare system and in health system governance? Active or passive? And what are implications? |
| **7.3 Role of the informal caregiver** | The informal caregiver is family, friends, neighbours etc. What is their role in the healthcare system and in health system governance? Active or passive? And what are implications? |
| **7.4 Linkage care sector and community** | How do they work together? |

References

1. World Health Organization. Monitoring the building blocks of health systems: a handbook of indicators and their measurement strategies. Geneva: World Health Organization; 2010 [1 April 2023]; Available from: https://apps.who.int/iris/handle/10665/258734.

2. Zürn M. Global governance as multi-level governance. In: Henrik E, Sonja W, Michael Z, editors. Handbook on Multi-level Governance. Cheltenham, UK • Northampton, MA, USA: Edward Elgar Publishing Limited; 2010.

3. Bigdeli M, Rouffy B, Lane BD, Schmets G, Soucat A. Health systems governance: the missing links. BMJ Glob Health. 2020;5(8). Epub 2020/08/14. DOI: 10.1136/bmjgh-2020-002533

4. Lehmann U, Gilson L. Action learning for health system governance: the reward and challenge of co-production. Health Policy Plan. 2015;30(8):957-63. Epub 2014/08/28. DOI: 10.1093/heapol/czu097

5. World Health Organization. Health systems governance. Geneva: World Health Organization; 2022 [1 April 2023]; Available from: https://www.who.int/health-topics/universal-health-coverage/health-systems-governance#tab=tab_1.

6. Mikkelsen-Lopez I, Wyss K, de Savigny D. An approach to addressing governance from a health system framework perspective. BMC Int Health Hum Rights. 2011;11:13. Epub 2011/12/06. DOI: 10.1186/1472-698X-11-13

7. World Health Organization. Everybody's business -- strengthening health systems to improve health outcomes: WHO's framework for action. Geneva: World Health Organization; 2007 [1 April 2023]; Available from: https://apps.who.int/iris/handle/10665/43918.

8. World Health Assembly. The World Health Report 2000: health systems: improving performance. Geneva: World Health Organization; 2000 [1 April 2023]; Available from: https://apps.who.int/iris/handle/10665/79020.

9. Minkman MMN. Suitable Scales; Rethinking Scale for Innovative Integrated Care Governance. Int J Integr Care. 2020;20(1):1. Epub 2020/01/15. DOI: 10.5334/ijic.5468

10. Provan KG, Kenis P. Modes of Network Governance: Structure, Management, and Effectiveness. Journal of Public Administration Research and Theory. 2008;18(2):229-52. DOI: 10.1093/jopart/mum015

11. Klijn E-H, Koppenjan J. Governance network theory: past, present and future. Policy & Politics. 2012;40(4):587-606. DOI: 10.1332/030557312x655431

12. Emerson K. Collaborative governance of public health in low- and middle-income countries: lessons from research in public administration. BMJ Glob Health. 2018;3(Suppl 4):e000381. Epub 2018/10/27. DOI: 10.1136/bmjgh-2017-000381

13. Torfing J, Peters B, Pierre J, Sørensen E. Interactive Governance: Advancing the Paradigm. Oxford, UK: Oxford University Press; 2012.

14. World health Organization. Voice, agency, empowerment: Handbook on social participation for universal health coverage. Geneva: World health Organization; 2021 [cited 1 April 2023]; Available from: https://www.who.int/publications/i/item/9789240027794.

15. Greer SL, Wismar M, Kosinska M, World Health Organization - Regional Office for Europe. Towards intersectoral governance: lessons learned from health system governance. Public health panorama. 2015;01(02):128-32. DOI: https://apps.who.int/iris/handle/10665/325467

16. Suter E, Mallinson S. Accountability for coordinated/integrated health services delivery. Copenhagen: WHO Regional Office for Europe; 2015 [1 April 2023]; Available from: https://www.euro.who.int/__data/assets/pdf_file/0003/286149/Accountability_for_coordinated_integrated_health_services_delivery.pdf.

17. Schmeer K. Guidelines for Conducting a Stakeholder Analysis. November 1999. Bethesda, MD: Partnerships for Health Reform, Abt Associates Inc.; 1999 [September 20, 2020]; Available from: https://www.who.int/management/partnerships/overall/GuidelinesConductingStakeholderAnalysis.pdf.

18. Gore RJ, Fox AM, Goldberg AB, Bärnighausen T. Bringing the state back in: Understanding and validating measures of governments' political commitment to HIV. Global Public Health. 2014;9(1-2):98-120. DOI: 10.1080/17441692.2014.881523

19. GAVI. What are the Health System Building Blocks? Project Fact Sheet No.5. 2013 [1 April 2023]; Available from: https://hssfactsheets.weebly.com/uploads/4/8/1/1/48110245/gavi_cso_fact_sheet_no_5_building_blocks.pdf.

20. Suter E, Oelke ND, da Silva Lima MAD, Stiphout M, Janke R, Witt RR, et al. Indicators and Measurement Tools for Health Systems Integration: A Knowledge Synthesis. Int J Integr Care. 2017;17(6):4. Epub 2018/03/29. DOI: 10.5334/ijic.3931

21. World Health Organization. Quality of care : a process for making strategic choices in health systems. Geneva: World Health Organization; 2006 [1 April 2023]; Available from: https://apps.who.int/iris/handle/10665/43470.

22. Harolds JA. Quality and Safety in Health Care, Part VI: More on Crossing the Quality Chasm. Clin Nucl Med. 2016;41(1):41-3. Epub 2015/10/09. DOI: 10.1097/RLU.0000000000001012

23. Stumm J, Thierbach C, Peter L, Schnitzer S, Dini L, Heintze C, et al. Coordination of care for multimorbid patients from the perspective of general practitioners - a qualitative study. BMC Fam Pract. 2019;20(1):160. Epub 2019/11/22. DOI: 10.1186/s12875-019-1048-y

24. World Health Organization, PEPFAR, UNAIDS. Task shifting : rational redistribution of tasks among health workforce teams : global recommendations and guidelines. Geneva: World Health Organization; 2007 [1 April 2023]; Available from: https://apps.who.int/iris/handle/10665/43821.

25. Chen L, Evans T, Anand S, Boufford JI, Brown H, Chowdhury M, et al. Human resources for health: overcoming the crisis. Lancet. 2004;364(9449):1984-90. Epub 2004/11/30. DOI: 10.1016/S0140-6736(04)17482-5

26. Leong SL, Teoh SL, Fun WH, Lee SWH. Task shifting in primary care to tackle healthcare worker shortages: An umbrella review. Eur J Gen Pract. 2021;27(1):198-210. Epub 2021/08/03. DOI: 10.1080/13814788.2021.1954616

27. van Schalkwyk MC, Bourek A, Kringos DS, Siciliani L, Barry MM, De Maeseneer J, et al. The best person (or machine) for the job: Rethinking task shifting in healthcare. Health Policy. 2020;124(12):1379-86. Epub 2020/09/10. DOI: 10.1016/j.healthpol.2020.08.008

28. Savigny Dd, Adam T, Alliance for Health Policy Systems Research, World Health Organization. Systems thinking for health systems strengthening / edited by Don de Savigny and Taghreed Adam. Geneva: World Health Organization; 2009 [1 April 2023]; Available from: https://apps.who.int/iris/handle/10665/44204.

29. Mounier-Jack S, Griffiths UK, Closser S, Burchett H, Marchal B. Measuring the health systems impact of disease control programmes: a critical reflection on the WHO building blocks framework. BMC Public Health. 2014;14:278. Epub 2014/03/29. DOI: 10.1186/1471-2458-14-278
