# Supplementary material for: Examining Macro-Level Barriers and Facilitators to Scaling Up Integrated Care from a Complexity Perspective: A Multi-Case Study of Cambodia, Slovenia, and Belgium
Source: Int J Integr Care. 2024 Nov 12;24(4):8. doi: 10.5334/ijic.7650 (PMC11568809; doi:10.5334/ijic.7650)
Supplement: Appendices. — Appendix 1 to 9. [file ijic-24-4-7650-s1.zip › ijic-7650_martens-s1/6501bade1ee10.docx]

## Appendix 8. Preliminary deductive analysis resulting in ICCC plus framework


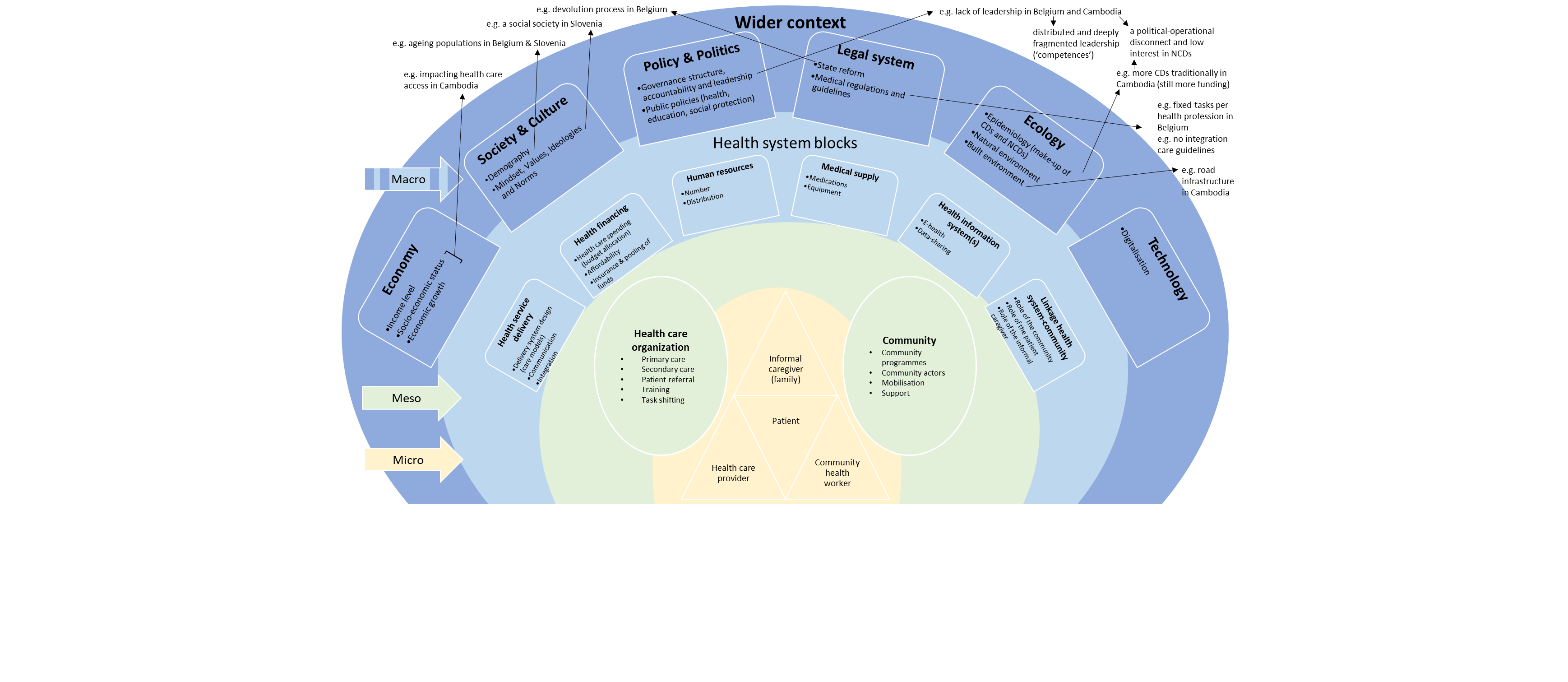


Note/legend: An initial attempt to develop a common codebook was to build on the WHO’s ICCC framework [1-5]. A preliminary deductive analysis was conducted by the first author (MM), resulting in an adapted ICCC, named the ICCC plus, which incorporated the WHO’s health system building blocks [6] and wider contextual factors beyond the health system (e.g. PESTEL: political, economic, socio-cultural, technological, environmental and legal).

**References**

1. Gilson L, Alliance for Health PS, Research - World Health Organization,. Health policy and systems research: a methodology reader / edited by Lucy Gilson. Geneva: World Health Organization; 2012 [1 April 2023]; Available from: https://apps.who.int/iris/handle/10665/44803.

2. World Health Organization - Health Services Delivery Programme; Division of Health Systems and Public Health. Integrated care models: an overview. Geneva: World Health Organization; 2016 [1 April 2023]; Available from: https://www.euro.who.int/__data/assets/pdf_file/0005/322475/Integrated-care-models-overview.pdf.

3. World Health Organization - Noncommunicable Diseases and Mental Health Cluster. Innovative care for chronic conditions : building blocks for actions : global report. Geneva: World Health Organization; 2002 [1 April 2023]; Available from: https://apps.who.int/iris/handle/10665/42500.

4. Epping-Jordan JE, Pruitt SD, Bengoa R, Wagner EH. Improving the quality of health care for chronic conditions. Qual Saf Health Care. 2004;13(4):299-305. Epub 2004/08/04. DOI: 10.1136/qhc.13.4.299

5. Nuno R, Coleman K, Bengoa R, Sauto R. Integrated care for chronic conditions: the contribution of the ICCC Framework. Health Policy. 2012;105(1):55-64. Epub 2011/11/11. DOI: 10.1016/j.healthpol.2011.10.006

6. World Health Organization. Monitoring the building blocks of health systems: a handbook of indicators and their measurement strategies. Geneva: World Health Organization; 2010 [1 April 2023]; Available from: https://apps.who.int/iris/handle/10665/258734.
