# Supplementary material for: Examining Macro-Level Barriers and Facilitators to Scaling Up Integrated Care from a Complexity Perspective: A Multi-Case Study of Cambodia, Slovenia, and Belgium
Source: Int J Integr Care. 2024 Nov 12;24(4):8. doi: 10.5334/ijic.7650 (PMC11568809; doi:10.5334/ijic.7650)
Supplement: Appendices. — Appendix 1 to 9. [file ijic-24-4-7650-s1.zip › ijic-7650_martens-s1/6501baf47eb3b.docx]

## Appendix 9. Comprehensive analysis table with selected quotes

| **Main and sub-themes** | **Cambodia** | **Slovenia** | **Belgium** |
| --- | --- | --- | --- |
| **1. Governance** | **Limited governance for NCDs** (in line with limited financial commitment from government and donors) | **Strong centralised governance with highly developed bureaucrac**y | **Fragmented, multi-level (partially decentralised) health governance** |
| 1.1 NCD policies and strategic plans on IC for chronic diseases | **NCDs outlined as prioritised area in Health Strategic Plan, yet the implementation is slow due to the absence of comprehensive guideline or NCD policy for implementation.** | **Many PHC reforms since 2016 [1] but human resources strategy is lacking. Stakeholders (HCWs, patients’ representatives) are not sufficiently involved in the development of strategies for organisation of long-term care.** | **Many pilots and high number of policy plans on IC for chronic diseases but no shared long-term vision.**  **Limited resources and time to support implementation and evaluate policies [2, 3].** |
| 1.2 Stakeholder collaborations (collaborative governance) | **Donor-driven**: implementation based on the availability of funding, following pre-defined objectives and indicators set by donor. | **Sub-optimal collaboration due to lack of communication between** **professional representatives** and **fragmentation of departments at MoH.** | **At federal level, the NIHDI concertation model is organised in silos:** (1) too many boards and councils, which slows down the decision-making process; (2) lack of representation of patients. |
| 1.2.1 Inter-sectoral approach | **Insufficient coordination and planning** *"Scaling-up T2D and HTN intervention requires multi-actor approach and we need to open space for their involvement as a complementary approach. We cannot do it alone and in a separate way." (Representative from NGOs)* | **Non-alignment of stakes of key organisations and stakeholders, resulting in coordination difficulties.** *Ma: "Local authorities, the Ministry of Health, public institutions and others play an important role in the decision-making process, and there are sometimes difficulties in linking them."* | **The multi-stakeholder negotiation model and multi-level governance make central level collaboration difficult, but reforms at decentral level are promising.** The sixth state reform caused fragmentation of public health governance competences, making inter-governmental collaboration more relevant but also more difficult [2, 3].  **Flemish primary care zones with their multisectoral care councils are a good example of intersectoral collaboration.** |
| 1.3 Accountability | **Negative influence of donor funding on the health policy process:** The donor’s ability to control indirect financial and political incentive might **decrease the MoH’s accountability and ownership.** Especially when the proportion of external funding exceeds the government expenses, priority-setting is affected. | **Strong state capability, but limited institutional capacity at the MoH to ensure effective implementation of National Health Plans since ownership at decentralised level.** *"PHC facilities are under the jurisdiction of municipalities, whose limited capacity and varying degree of economic prosperity have a number of negative implications for their performance [...]. The Ministry of Health has no power to address this issue, because of its lack of line authority over CHCs. The absence of line authority over the PHC facilities is compounded by the absence of any other accountability mechanism."* [4] | **Negatively influenced by fragmented governance:** In view of the complexities in political structure and partial overlapping responsibilities, the governments themselves sometimes do not know what the rules exactly are and what they are responsible for. The multi-stakeholder model (Medicomut) allows input of government, health financing organisations, providers and users, but the negotiation processes are not transparent and influenced by power.  ***"****Who is responsible for what?* *What does it all mean? One decree says this, the policy vision lays down the nuances somewhat differently. We repeatedly insisted on a clarification of the various issues involved, but we already had the feeling that Flanders didn't really know." (IV15)* |
| 1.4 Leadership | **Political leadership: low. Organisational leadership at sub-national level: variable.** *"Some health facility managers do not have a leadership capacity and do not know what it takes to become a leader themselves." (Representative from MOH)* | **Political leadership: volatile Organisational leadership: high (bottom-up)** Frequent turnover of key stakeholders among decision-makers (due to elections, extra elections, resignments of governments etc.), who are therefore unable to make long-term strategies for system change. | **Political leadership: fragmented, lacking (2019) Organisational leadership: medium** (since multi-sectoral coordination - key for IC - is in conflict with organisation culture: self-employed, high autonomy in organising their own general practices within PHC). *“integration on macro level is missing”; “there is no integrated politics” (bureaucrat, civil servant)* |
| 1.5 Political interest and power dynamics | **Infectious diseases still dominant in resource allocations and health system structures and actors.** | **Volatile political environment due to high turnover of governments (see 1.4), which means certain big reforms are halted and changes to make the health system more sustainable are delayed.** | **Struggle and slowdown in** **decision-making due to negotiation model and varying ideological interests across decision-making levels (preferences due to 9 MOHs)**. Some HCWs fear reform, as they do not want to lose power, resources, roles or status. The professions with the most to lose have greater power are more strongly represented in decision-making structures. |
| 1.6 Political commitment | **Low:** reflected in the limited financial commitment of the government to NCDs. | **Low:** The high turnover of political leaders goes hand in hand with **political uncertainty**. Thereby policy development and implementation for IC can be slowed down. | **Low:** (a) IC (pre-COVID-19) is **not a high-profile public issue,** which means politicians are not held accountable; (b) **the required changes might contrast with the political preference** of the decision-makers that are often contingent on ideological preference. |
| **2. Health service delivery** | Lack of UHC. The scale-up of quality NCD care is constrained due to other structural barriers: lack of HRH, financial resources, infrastructure, and medical supply. | Near UHC and fairly integrated service delivery due to multi-profile teams. PHC in Slovenia is based upon the vision of “comprehensive, integrated, accessible” care that is “affordable for everyone and everywhere.” [4] | Near UHC. Collaboration in interprofessional teams largely uncommon. |
| **2.1 Structural** |  |  |  |
| 2.1.1 Public-private mix | **Mix of services:** - **Low utilization of public health services** [5].  - **High utilization of the private health services might cause a higher proportion of OOP spending that could lead to catastrophic health expenditure.**  *"A majority of Cambodian people (60%) with chronic diseases are diagnosed and treated in private facilities."*[6] | **Mix of services:**  **- Increase in private providers** *"(…)* *recent years have seen an increase in the number of private providers. This has led to complex contracting arrangements and a degree of fragmentation in service provision."* [4]  *MiP: "****healthcare is becoming commercialised*** *and more and more medicines and services have to be paid for. Doctors are hard to find and some have to turn to private practices, which are expensive."* | **Privatised, but regulated:**  Most HCWs in Belgium are self-employed, the repercussion of their therapeutic freedom and entrepreneurship is that it is difficult to regulate organisation and practice. *'"The provision of care is based on the principles of independent medical practice, (…). Reimbursed healthcare services are provided by both public and private institutions and individual healthcare providers who mainly comply with the same set of rules, enjoy the same therapeutic freedoms and offer the same services."* [7] |
| 2.1.2 Referral system | **Poor referral system in ensuring continuum of care** from community to HC or from HC to RH.  *"Currently, there are problems with referral of patients from HC to RH which we cannot track if they are actually arrived the referral hospitals after being referred." (Representative from MoH)* | **GPs act as gatekeepers** to specialist care. Communication between primary and secondary/tertiary care is mainly through paper (referral by GP and medical report by primary/secondary HCW). *"The participants pointed out that referrals and medical reports are systematically organized but often not entered to the electronic records." [8]* | Patients are free to consult any GP or specialist, therefore **GPs do not play a gatekeeping role**. Because healthcare is based on the free choice of physician and mainly on the fee-for-service payment, the challenge lies in the fact that a **fee-for-service model does not stimulate cooperation and referral,** since referring a patient to somebody else means potentially cutting the health worker’s own income. |
| 2.1.3 Geographical coverage | **HC coverage with WHO-PEN is still limited**: 31 NCD clinics at RHs out of a total of 125 RHs (24.8% coverage), 121 HCs of HC-based care out of a total of 1,221 HCs (10% coverage) within 23 ODs of a total of 102 ODs in the whole country (22.5% coverage). | **Fairly universal geographical coverage.** | **Good geographical coverage, noting that 98% of Belgium is considered urban.** |
| **2.2 Organisational** |  |  |  |
| 2.2.1 Quality of service | **Low in public facilities:** (a) Perceived lower service quality compared to private facilities; (b) Quality of services depends on cross-cutting issues; lack of supply, competent staff or materials etc. | **High, yet:** Change in the organization and adjustments of responsibilities at PHC level had an impact on quality of care: increasing competences of GPs led to increasing workload without reinforcing support mechanisms. Therefore, GPs cannot provide optimal care. | **High** *"In general, the Belgian population has good access to* ***many high-quality health services****."* [7] |
| 2.2.2 Supervision and monitoring | **Lack of supervision for HCWs on how to implement WHO-PEN.** **Private provision not well regulated by MOH.** | **Assigned institutions for oversight, but no clear lines of accountability, hence the role of the MoH as steward could be strengthened**. | **Regular, but minimal and not tailored to IC. Efforts to promoting and developing population health management ongoing.** |
| 2.2.3 Coordination of care | **Current implementation of IC does not specify the role or function of care coordinator** to liaise people with NCDs with different health providers. | **Informally and sub-optimally, GP mostly acts as care coordinator.** Health service providers at different levels are not coordinated in their treatment of patients, due to lack of communication, resulting in doubling of services [8]. | **Informally and sub-optimally, GP usually acts as care coordinator, but debate ongoing about this, how to best organise the coordination of care**. The debate and implications of the outcome link to corporatism; everyone wants to take on the role of coordinator, as it reinforces the importance of a certain HCW role or function. |
| 2.2.4 Access to care (to integrated PHC) | **Road infrastructure and/or distance (transport fee and companion to health facility) as barrier to accessing healthcare for some patients.** | **Difficulties in accessing PHC (CHC) services at primary level, because of the shortage in staff; and waiting times at secondary and tertiary level** | **Despite fairly UHC, out-of-pocket payments remain high and important part of population postpones healthcare for financial reasons** |
| 2.2.5 Patient/person centeredness | **Importance of involving family members and community resources in patient care.** | **Patient-centeredness is a priority for the Government; patients have many rights, patient centeredness in care is important.** | **Person centred care has been a buzzword in recent years, but it hasn't been put in practice.** Many agree the current system does not put the person in the centre yet. A culture change will be needed. |
| 2.2.6. Continuum of care (including role of preventive programmes) | **Lacking continuity of care, patients are lost in the system / not tracked (after referral).** | **Good continuity of care, national Preventive Programmes are well organized, HECs are considered key but attendance/referral needs to be stimulated**. | **Continuity of care, yet some patients fall through the gaps. Only a small budget for prevention and a high share of preventable hospitalisations. Moreover, FFS does not stimulate referral.** |
| 2.2.7 Interprofessional teams | **In PHC, there is a multi-professional team, but most dominant are nurses and mid-wives. CHWs (VHSGs) have been an extension of HCs for some out-reach works at the community level. So collaboration of CHWs with health centres is common.** | **In PHC, multi-profile teams work together.** | **In primary care, interprofessional teams only rarely exist**, **although they are rising. Mostly primary care practices exist only of GPs**. |
| 2.2.8 Organisation culture | Strong hierarchy (only physicians can provide treatment). | **Multi-disciplinary teams within PHC, successful in integrating public health services into PHC**  - in accordance with its socialist system and the alma atta declaration). - yet fragmented communication between PHC and specialised care.  - strong hierarchy (GPs are the decision maker) [9]. | **Strong, highly specialised, supply-oriented system, in which therapeutic freedom and entrepreneurship are important, also in the organisation of care. There are many solo practices.** *"Most GPs work in solo practice, although group practices are gaining popularity among newly graduated GPs. Community health centres with capitation based remuneration are also gaining popularity."* [7] |
| **3. Health financing** | Limited NCD funding and coverage and high OOP | Outdated cost model | FFS provider payment system directly impeding IC |
| 3.1 Budget/ funding for IC (collection of funds) | **Limited funding for NCD programs:**  NCDs account for only 21% of total health spending [10]. *"Government budget is not enough (…). However, external donors cannot guarantee a long-term [sustainable] work." (Representative from MoH)* | **The health budget is constrained, with some reporting inefficient allocation of resources** *"Health expenditures in Slovenia are at 8.5% and is below EU-average (10%)."* [11] | **Too much inefficient spending (in specialised care, when care could also be delivered on a lower level, in PHC).** |
| 3.1.1 Service user financial payment (including out-of-pocket) | **Limited healthcare scheme to refund healthcare expenses to users.** **OOP remains the top contributor of health spending:**  OOP is around 60% in 2016, which is also the case for NCDs [10]. | **One of the lowest OOPs in EU (11.7 % of health spending)**, **yet OOP is an important barrier to access and related to commercialisation, i.e. the increase in private providers (**48,3% OOP payments as % of private expenditure on health) [11]*.* | **OOP is on the rise**. The share of health spending paid directly by households is still 18%—a higher share than that in the EU overall (15%) [12]. |
| 3.2 Social health protection (risk pooling) | **Coverage of Social Protection Scheme is still low compared to total population** (38.5% covered in 2019) [13]. | **Access to services covered by compulsory social health insurance** [14]  *Ma: "there is insufficient information on the distribution of funds, the distribution of funds is inadequate."* | **Compulsory social health insurance:** 99% of the population for a broad package  Prices of healthcare utilisation can vary while refunding of cost is same regardless of expense. This might lead to less protection in case of increased (‘deconventional’) pricing by providers. |
| 3.3 Service provider financial payment (purchasing and resources allocation) | **Mixed health financing, narrow streams of funding, OOP important** (especially to pay at private facilities). Public providers mainly funded by government budget (salary). Also by user fees, reimbursement from case-based payment, service delivery grants (performance‐based and fixed lump sum grants). User fees (OOP) are complementing the low-paid health personnel. | **Outdated cost model.**  Primary healthcare providers are paid through a combination of capitation and fee-for-service. The reimbursements for healthcare providers are calculated upon data from several years ago, without corrections for economic growth, inflation etc., which results in the outdated cost model. | **The FFS provider payment mechanism system hinders care from becoming integrated:** the predominant FFS system incentivizes each individual service, or, acute care provision. Chronic care requires multiple other tasks, such as cooperation, referral, follow-up and coordination, which are currently not reimbursed. |
| **4. Health workforce** | Shortages and low trust in quality of care at public primary care facilities. Moonlighting and brain drain to private sector. | PHC providers are overburdened, dissatisfied, periodically threatening to strike or resign [4]*.* | Increasing challenge: less GPs, training and organisation to facilitate IC and regulations impeding task shifting to lower cadres. |
| 4.1 Availability / shortages | **Shortages of healthcare staff in all areas of public health facilities** **exist, due to the lack of incentives.** Limited qualified health human resources for NCDs are hindering the speed of scaling up NCD care provision within PHC. | **Slovenia has one of the lowest numbers of GPs in Europe (below EU average), but number of nurses is higher than EU average** [11]**. Moreover GPs are overworked and underpaid, compared to European countries.** | **Low number of GPs (yet above EU average). Challenges are that many of them are aged and will retire soon and that the** **number of full-time equivalent GPs is expected to decrease** **slightly**. **Number of nurses are high (above EU average), but low number of them work in PHC** [12]. |
| 4.2 Human resource planning/management | **Lacking the supplies and capacity to implement diagnosis and treatment of NCDs at HCs** (related to funding issues and lacking policy on NCD). | **Strategy on HRH (management) lacking**  (see theme 1.1) | **Planning model in place.** |
| 4.3 Training | **Lack of training in NCD care provision to HC staff. Absence of leadership and management training to key staff.** | **Regulated and focused on a strong PHC system.**   - **Highly trained in team collaboration.** - **Yet, migrant HCWs are less regulated**. | **Training focused on clinical management with specialist focus. Topical course on interprofessional collaboration, but less on organising IC and team work.** |
| 4.4 Task shifting | **Limited HRH lead to high staff turnover affecting competences necessary for task delegation. Potential role for the CHW (see more: 7.4).** | **Seen as strategy to respond to HRH shortages (GPs in particular), the increasing BoD and number of tasks:** - Certain competences (some simple medical conditions and procedures) could be delegated to nurses, pharmacies, and community (lay educators). - Already increased role of nurses in PHC to manage patients with NCDs. | **Seen as strategy to respond to current challenges (cf. Slovenia) and also to adapt to patient needs, yet made difficult due to organisational culture and existing regulation**.  Efficient division of tasks is blocked by the law on the execution of tasks by healthcare providers as well as by reimbursement rules, as some procedures are only remunerated when performed by a physician. |
| **5. Medical supply** | Shortages | Well-resourced | Well-resourced |
| 5.1 Medications (supply and availability) | **Medicine supply for NCDs is insufficient for routine operation at HCs.** | Access to all modern treatment options is available. | **Supply and availability of medications is sufficient for all types of NCDs. A 'home pharmacist' was introduced to help patients with polypharmacy to manage their medication.** |
| 5.2 Equipment (supply and availability) | **Materials are supplied only at the beginning of the intervention tackling NCDs, e.g. WHO-PEN at HCs. There is no monthly supply or continuous supply.** This limits the sustainability of implementation. | Access to all modern treatment options is available. | **Equipment for chronic diseases, such as laboratory test are available, also at primary care level.** |
| **6. HIS** | Fragmented and weak | Fragmented | Fragmented |
| 6.1 E-health / digital health | **A plan for digital health is developed, yet data management is a problem due to a lack of integration of information (no NCD info and PMRS in the HMIS),** **lack of HCWs, poor internet services and lack of a web-based system.** | **Some national eHealth projects are running efficiently, but shortcomings in the design of the eHealth system exist** *"to improve the performance of their PHC systems, they will need to (dramatically)* ***improve the user friendliness, functionality and interoperability of their electronic patient record systems****"* [4]*.* | **Debate on role of government vs. market in the provision of software:** disagreement at decision-making level about whether to pursue a common, centrally governed system for the entire healthcare system or to further stimulate the role of private companies competing on the free market. Problems in interoperability of systems. |
| 6.2 Data availability, registration & monitoring of clinical care data | **Limited health facility-based data and no major database for NCDs.** | **Registering patient data in the digital HIS seen as time consuming, hence comprehensive digital patient data limited, despite central collection. Population management is impeded.** | **Registering patient data in the digital HIS seen as time consuming, hence comprehensive digital patient data limited. Population management is impeded.** |
| 6.3 Fragmentation of data sources & data sharing between healthcare professionals and across levels | **Data available in different sources (project-based), unable to merge in one database.** | **Too many different software; data not systematic; lack of connectivity of patient data between levels** *"Because every municipality [as owner of the CHCs] and each hospital procure their own IT-systems from a variety of private software companies, none of the clinical records are directly linked. (…) Information sharing among providers at the different levels of care is restricted."* [4] | **Different software packages by a variety of commercial players that cannot link or exchange data with other relevant systems of medical professional groups. Patients and the social sector are completely disconnected from the platforms used to share information. There are also privacy concerns and unclear legislation.** |
| **7. Link health system-community (demand)** | Focus on strengthening the role of CHWs as well as HCWs at HCs | Focus on low-level care and task delegation (towards more emphasis on peer support) | Focus on strengthening link between PHC and social sector and increasing patient, social worker and local government representation and their roles in coordination of PHC. |
| 7.1 Role of community actors | **Important role of CHW in chronic disease management, due to trust and closeness to patients in the community, yet not yet well supported and incentivised.** | **Fragmentation informal associations in the community (yet also professional and reliable)**. | Social workers (from the social welfare sector) get **no access to any patient data and no incentive to collaborate with HCWs**, except a seat in the care council within primary care zones. |
| 7.2 Role of the patient / public | Patient voice limited/absent in decision-making. | **Patient associations are fragmented and insufficiently funded [9]***.* **Patient voice in the evaluation and planning not consistent**. | **Various patient organisations. In policy, person-centeredness popular term, possibly tokenistic, due to the often passive position of the patient in practice and dominant supply orientation. Patient representation is limited in official decision making (e.g. Medicomut), but rising, such as in the primary care zones.** |
| 7.3 Role of the informal caregiver | **Important role, due to trust and support, but only informally involved** whilst providing self-management support, social care, transportation and acting as a companion to health facilities. | **There is a need to integrate informal caregivers in the system (due to trust and BoD).** | **Role for informal caregivers in primary care council in primary care zones (in Flanders), not strongly represented otherwise.** |
| 7.4 Linkage healthcare organisation and community | **Position of CHW could be further strengthened, to empower them to take up a liaison role between the people at home, the community, and healthcare providers.** Issues relate to technical and financial support, and hierarchy in relation to healthcare professionals. | **Efforts on moving care closer to the patients and their home, to increase link between healthcare and community.** | In primary care zones in Flanders and Brussels, its care councils have various **community as well as HCW representatives**. Link challenged due to **varying incentive systems between medical and social sector** (e.g. FFS vs. salary) cause difficulties in collaboration. |

*Note: The following abbreviations are used in the table: BOD = burden of disease, CHC = community health centre, CHW = community health worker, GP = general practitioner, FFS = fee-for-service, HC = health centre, HCWs = healthcare workers, HECs = health education centre, HRH = human resources for health, IC = integrated care, MoH = Ministry of Health, OOP = Out-of-pocket expenditure, PCZ = Primary Care Zones, PEN = Package of Essential NCD interventions (implemented in Cambodia), PHC = primary healthcare, RH = referral hospital, THE = total health expenditure, UHC = universal health coverage, WHO = World Health Organization*

**References**

1. European Observatory on Health Systems and Policies. Slovenia: health system review 2021. 2021 [1 April 2023]; Available from: https://eurohealthobservatory.who.int/publications/i/slovenia-health-system-review-2021.

2. Danhieux K, Martens M, Colman E, Wouters E, Remmen R, van Olmen J, et al. What Makes Integration of Chronic Care so Difficult? A Macro-Level Analysis of Barriers and Facilitators in Belgium. Int J Integr Care. 2021;21(4):8-. DOI: 10.5334/ijic.5671

3. Martens M, Danhieux K, Van Belle S, Wouters E, Van Damme W, Remmen R, et al. Integration or Fragmentation of Health Care? Examining Policies and Politics in a Belgian Case Study. Int J Health Policy Manag. 2021;11(9):1668-81. Epub 2021/07/19. DOI: 10.34172/ijhpm.2021.58

4. World Health Organization - Regional Office for Europe. Integrated, person-centred primary health care produces results: case study from Slovenia. Copenhagen: World Health Organization; 2020 [1 April 2023]; Available from: https://apps.who.int/iris/handle/10665/336184.

5. Chhim S, Te V, Buffel V, van Olmen J, Chham S, Long S, et al. Healthcare usage and expenditure among people with type 2 diabetes and/or hypertension in Cambodia: results from a cross-sectional survey. BMJ Open. 2023;13(1):e061959. DOI: 10.1136/bmjopen-2022-061959

6. Bigdeli M, Jacobs B, Men CR, Nilsen K, Van Damme W, Dujardin B. Access to Treatment for Diabetes and Hypertension in Rural Cambodia: Performance of Existing Social Health Protection Schemes. PLOS ONE. 2016;11(1):e0146147. Epub 2016/01/28. DOI: 10.1371/journal.pone.0146147

7. Gerkens S, Merkur S. Belgium: Health system review. Health Syst Transit. 2010;12(5):1-266, xxv. Epub 2011/01/13. DOI:

8. Klemenc-Ketis Z, Stojnic N, Zavrnik C, Ruzic Gorenjec N, Danhieux K, Lukancic MM, et al. Implementation of Integrated Primary Care for Patients with Diabetes and Hypertension: A Case from Slovenia. Int J Integr Care. 2021;21(3):15. Epub 2021/10/26. DOI: 10.5334/ijic.5637

9. Zavrnik C, Danhieux K, Monarres MH, Stojnic N, Lukancic MM, Martens M, et al. Scaling-up an Integrated Care for Patients with Non-communicable Diseases: An Analysis of Healthcare Barriers and Facilitators in Slovenia and Belgium. Zdravstveno varstvo. 2021;60(3):158-66. Epub 2021/07/13. DOI: 10.2478/sjph-2021-0023

10. World Health Organization - Regional Office for the Western Pacific. Cambodia national health accounts (‎2012-2016)‎: health expenditure report. Geneva; 2019 [cited World Health Organization 1 April 2023]; Available from: https://apps.who.int/iris/handle/10665/325903.

11. OECD/European Observatory on Health Systems and Policies. Slovenia: Country Health Profile 2021. OECD; 2021 [1 April 2023]; Available from: https://www.oecd-ilibrary.org/social-issues-migration-health/slovenia-country-health-profile-2021_1313047c-en.

12. OECD/European Observatory on Health Systems and Policies. Belgium: Country Health Profile 2021. OECD; 2021 [1 April 2023]; Available from: https://www.oecd-ilibrary.org/social-issues-migration-health/belgium-country-health-profile-2021_57e3abb5-en.

13. Ministry of Health - Department of Planning and Health Information - Cambodia. Annual Health Sector Progress Report 2019 and Way Forwards for 2020. 2020.

14. Bohm K, Schmid A, Gotze R, Landwehr C, Rothgang H. Five types of OECD healthcare systems: empirical results of a deductive classification. Health Policy. 2013;113(3):258-69. Epub 2013/10/08. DOI: 10.1016/j.healthpol.2013.09.003
